# Expanding the Horizons of Machine Learning in Nanomaterials to Chiral Nanostructures

**DOI:** 10.1002/adma.202308912

**Published:** 2024-02-03

**Authors:** Vera Kuznetsova, Áine Coogan, Dmitry Botov, Yulia Gromova, Elena V. Ushakova, Yurii K. Gun’ko

**Affiliations:** School of Chemistry, CRANN and AMBER Research Centres, Trinity College Dublin, College Green, Dublin D02 PN40, Ireland; School of Chemistry, CRANN and AMBER Research Centres, Trinity College Dublin, College Green, Dublin D02 PN40, Ireland; Everypixel Media Innovation Group, 021 Fillmore St., PMB 15, San Francisco, CA 94115, USA, Neapolis University Pafos, 2 Danais Avenue, Pafos 8042, Cyprus; Department of Molecular and Cellular Biology, Harvard University, 52 Oxford St., Cambridge, MA 02138, USA; Department of Materials Science and Engineering, and Centre for Functional Photonics (CFP), City University of Hong Kong, Hong Kong SAR 999077, P. R. China; School of Chemistry, CRANN and AMBER Research Centres, Trinity College Dublin, College Green, Dublin D02 PN40, Ireland

**Keywords:** artificial intelligence, chirality, machine learning, nanomaterials, nanoparticles

## Abstract

Machine learning holds significant research potential in the field of nanotechnology, enabling nanomaterial structure and property predictions, facilitating materials design and discovery, and reducing the need for time-consuming and labor-intensive experiments and simulations. In contrast to their achiral counterparts, the application of machine learning for chiral nanomaterials is still in its infancy, with a limited number of publications to date. This is despite the great potential of machine learning to advance the development of new sustainable chiral materials with high values of optical activity, circularly polarized luminescence, and enantioselectivity, as well as for the analysis of structural chirality by electron microscopy. In this review, an analysis of machine learning methods used for studying achiral nanomaterials is provided, subsequently offering guidance on adapting and extending this work to chiral nanomaterials. An overview of chiral nanomaterials within the framework of synthesis–structure–property–application relationships is presented and insights on how to leverage machine learning for the study of these highly complex relationships are provided. Some key recent publications are reviewed and discussed on the application of machine learning for chiral nanomaterials. Finally, the review captures the key achievements, ongoing challenges, and the prospective outlook for this very important research field.

## 1. Introduction

In recent years, the huge surge in interest and use of machine learning (ML), has led to transformations in a wide range of fields, increasingly reshaping and changing how society tackles more modern and complex problems, including its use in powering speech recognition tools, self-driving vehicles, healthcare, and many other areas. The wide-ranging impact of ML continues to push the boundaries of scientific research and discovery, driving innovations that will shape our future for centuries to come.

ML first emerged as a concept in the 1950s, with the coining of the term attributed to Arthur Samuel, an IBM scientist who pioneered one of the earliest artificial intelligence (AI) tools—a computer program capable of mastering the game of checkers to the point where it could beat the average player.^[1]^ The field of ML has made remarkable progress in the years since, as evidenced by the literature, with an extensive body encompassing close to 640,000 works, with over 118,000 in 2023 alone, according to Scopus. Furthermore, these rapid advances in ML, and more generally AI, have been highlighted in a recent report from the Institute for Human-Centered Artificial Intelligence at Stanford University.^[2]^ The rapidly growing interest of the scientific community in ML approaches is also reflected in the increasing appearance of journals with a key focus on ML application in different fields of science, including “Proceedings of Machine Learning Research” “NPJ Computational Materials,” “Nature Machine Intelligence,” and “Digital Discovery,” to name a few. Furthermore, interested readers may refer to several recent reviews on the application of ML in different research directions of materials science.^[3–5]^ AI, and more specifically ML, are exciting prospects for improving the quality and speed of the development of materials science and, in particular, chiral nanomaterials (NMs). This is reflected in the sizeable number of recent reviews and perspectives which are devoted to the discovery of novel NMs, for example 2D materials,^[6]^ plasmonic NMs,^[7]^ carbon-based NMs,^[8]^ and beyond.^[9–11]^

The use of AI has been also shown to help in understanding the complex interconnections between the properties of NMs and their impact in various applications, ranging from photonics^[12]^ to nano-enabled agriculture.^[13]^ For instance, in a recent review on nanophotonics, the authors highlighted that deep learning models at present can effectively “learn” Maxwell’s equations by approximation, allowing for optical responses to be obtained without explicitly solving the equations. This allows researchers to discover and explore solutions beyond the existing knowledge and available data, enabling us to transfer these solutions to diverse sets of problems.^[14]^

The significance of employing ML for processing and analysis of data relating to NMs safety and biomedical applications has previously been emphasized elsewhere—interested readers may refer to the cited literature.^[15–18]^ For instance, in a recent review from Singh et al., a toolbox of ML methods was proposed for understanding bio-physicochemical identity for NMs applied in bio-applications, including the prediction of nano-bio interactions.^[17]^ In another recent review from Feng et al., the authors showed that AI may help to predict the immune response or immunotherapy effects of new NMs together with filling the gaps in the datasets.^[18–20]^ The implementation of ML methods in the environmental risk assessment of NMs in the realm of sustainability is also gaining considerable traction.^[21]^

ML is a powerful tool for finding common patterns and trends in a given dataset, classifying the data, and predicting results based on the available data. Instead of using a human-developed algorithm to solve a problem, the machine uses statistical methods to find its own algorithm based on solutions to similar problems. Perhaps the main task that can be solved using ML in the field of NMs research is identifying the relationship between the synthetic parameters, structure, and composition of the obtained materials, and their properties and applications.^[22]^ ML models can predict the structure and properties of the resulting products based on synthetic conditions, and can also solve the inverse problem of synthetic design of materials with desired properties. ML is a powerful tool for searching for materials with particular combinations of properties for specific applications, as well as for finding new utilizations for multifunctional materials. ML has also been proven to be an effective tool for experimental data analysis of NMs, particularly in the area of microscopy image classification. Solving problems using ML methods can often be much faster, cheaper, and more accurate than doing conventional experiments in a laboratory or using more traditional computer simulation methods.

Chiral NMs hold a significant and crucial place in the realm of nanoscience research, perhaps most significantly in the fields of biology and medicine. This phenomenal importance arises due to the fact that the chirality of a molecule, i.e., its molecular asymmetry, has a tremendous impact on its biochemical activity and toxicity.^[23,24]^ Most drug compounds are chiral, hence the production and analysis of the desired enantiomers of drugs are of great importance in the pharmaceutical industry. Therefore, there is a tremendous need for enantiomeric catalysts, adsorbents, and sensors, with chiral NMs emerging as promising candidates for fulfilling these roles. In addition, chiral NMs, due to their ability to selectively absorb circularly polarized light, are used in polarized optics, including three-dimensional (3D) vision.^[25]^ In the most obvious cases, chiral particles may have an asymmetric crystal lattice or geometric shape. However, even achiral nanocrystals (NCs) can acquire chiroptical properties when enantiomers of organic molecules are adsorbed on their surface or when achiral particles are assembled in an asymmetric manner.^[26]^ The NC, in turn, can influence the chiral properties and biochemical activity of the adsorbed chiral molecule.^[27]^ In addition, during any synthesis, spontaneous asymmetric defects in NCs can form, so the NM can have a certain number of chiral particles in a racemic ratio.^[28]^ This is important to take into account when using any NMs, either in biochemistry and medicine or indeed in any other fields where NM exposure is high since the chirality of the material significantly influences its toxicity.

The study of chiral materials will undoubtedly benefit significantly from the use of ML in the same way as the investigation of NMs in general, by significantly reducing the labor-intensive and time-consuming laboratory work and computer modeling, greatly increasing the sustainability of materials, and facilitating the promotion of materials into commercial and real-world applications. In particular, the determination of nanoparticle (NP) chirality using electron microscopy stands to benefit hugely from the more widespread implementation of ML, as it is widely used as a tool to identify patterns in images.^[29]^ However, it is important to highlight that, in contrast to their achiral counterparts, there is a notable scarcity of studies on the use of ML for chiral NMs.

Therefore, this review aims, along with a description of the latest achievements in ML-based studies of both achiral and chiral NMs, to summarize the experience of using ML for NM analysis and to provide insights into the possibility of transferring this knowledge and techniques to the study of chiral NMs. In this review, we introduce the concept of ML and an overview of ML methods and tasks that can be solved in materials science (Section 2). The main approaches of ML in the field of nanotechnology are discussed in Section 3, including synthesis–structure–property relationships, NM forward design, and inverse engineering. Section 4 is focused on the usage of ML for the investigation and development of NMs and NM-based devices for application in catalysis, electronics, and medicine. A discussion on chiral NMs is presented in Section 5, with Section 6 focusing on the use of ML for the field of chiral NMs—an emerging and developing field. Finally, the review culminates with an overview that summarizes potential future advancements and innovative approaches in applying ML for the further development of chiral NMs.

## 2. What Is Machine Learning?

Prof. Tom M. Mitchell, a founder of the Machine Learning Department at Carnegie Mellon University, and a pioneer of the field, provides the definition for ML: “A computer program is said to learn from *experience E* with respect to some class of *tasks T* and *performance measure P*, if its performance at *tasks in T*, as *measured by P*, improves with *experience E*.”^[30]^ The *performance* of a ML model measures “how good” it is at solving a particular task. To evaluate the performance of the model, we need to define one or more measurable parameters, such as accuracy metrics, depending on the type of task, and our understanding of what “good” is.

ML and traditional programming have fundamentally different approaches to data analysis. As researchers tackle more complex problems, the performance of the ML algorithm increases not due to an increase in the number of instructions in the program module, e.g., the number of conditions, cycles, subprograms, etc., but by increasing both the quality and amount of data in a dataset. This allows the ML approach to be much more flexible in considering the characteristics of the data, and to produce increasingly accurate results with growing data volume rather than programmed heuristics. Moreover, the application of ML offers a faster method for drawing conclusions from scientific data compared to conducting laboratory experiments. In practice, the use of ML is an effective method of overcoming the growing complexity of software systems, including those for the analysis of NMs.

With the aid of ML methods, materials scientists can solve the following problems: analysis of research papers, including text recognition; data mining and pre-processing; classification of data; analysis of data, including improving data quality and deriving dependencies in a given dataset; prediction of material structure and parameters; prediction of new materials and synthetic procedures based on a given target application.^[31]^

Depending on the type of problem being solved, the following ML approaches can be distinguished:

- *Supervised learning*. A model is trained to predict the answer on labeled data, i.e., in the training dataset, we know the correct target values. Here, we distinguish two main tasks: a classification, where the discrete target variable is a label of class from a finite set; and a regression, where the target variable values are continuous numeric scores.^[32]^
- *Unsupervised learning*. A model is trained to highlight patterns in data without labeling, for example, to perform a cluster analysis or highlight some patterns or anomalies in the data.^[33]^
- *Semi-supervised learning*. A combination of a small amount of labeled data and a large amount of unlabeled data is used to train models.^[34]^
- *Reinforcement learning*: A model is trained with set strategies, “game rules,” based on rewarding desired behaviors and/or punishing undesired ones.^[35]^

Deep learning (DL) is a subset of ML that employs algorithms called deep neural networks (DNN), which mimic the structure and functions of the human brain to analyze and process large datasets, identifying complex patterns and dependencies that are not obvious, or difficult to detect with traditional methods.^[36]^ These algorithms are capable of self-learning and adapting, improving their performance as they analyze more data.

DL can be employed using either standard training data extracted from an existing dataset, or by transfer learning, which is an incredibly powerful tool, particularly in domains with limited data. The transfer learning approach consists of a DNN, based on a transformer architecture, and pre-trained on large data, such as images or text sequences from a diverse range of subject areas.^[37]^ The pre-trained model can then be fine-tuned on a smaller, task-specific dataset, a process that often yields more accurate results compared to training a neural network from scratch on a limited data set. This is especially beneficial in the field of NMs, where data scarcity is a common challenge.

Currently, the main application areas of DL predominantly use transfer learning. Recent advancements in transfer learning have significantly impacted vision transformers and large language models. Vision transformers are often pre-trained on extensive datasets like ImageNet, and then subsequently fine-tuned for specialized tasks, proving efficient in areas with limited data.^[38]^ For large language models, such as GPT and BERT, extensive pre-training on vast text corpora and low-resource languages enables deep language understanding^[39]^ and adaptability to specialized domains in materials science.^[40,41]^ Notably, large language models and multimodal vision-language models (such as GPT-4 V, LLaVA) exhibit advanced capabilities in two novel approaches, namely zero-shot and few-shot learning.^[42–44]^ Zero-shot learning is a DL approach where a model performs tasks it has never explicitly been trained to do, using a deep understanding derived from its initial comprehensive training. Few-shot learning involves the model quickly adapting to new tasks with minimal additional examples. These approaches enable the performance of tasks with little to no additional training, marking a significant evolution in transfer learning efficiency. For materials scientists, these techniques offer the potential to analyze and predict properties of novel materials with very limited or no prior data on similar materials.

Transfer learning is currently underutilized in the study of NMs. However, its future potential as a valuable, useful tool for NM analysis is noteworthy, as transfer learning is highly effective in the analysis of small datasets, addressing one of the main problems in using ML for NMs—limited data. However, it is important to acknowledge that in some DL applications, especially those with ample task-specific data, training a model from scratch might be more appropriate. Therefore, the choice between transfer learning, zero-shot, and few-shot learning by large neural networks (NNs), or training from scratch, as well as other DL approaches, should be guided by the specific requirements and data availability of the task at hand.

**Figure 1.**
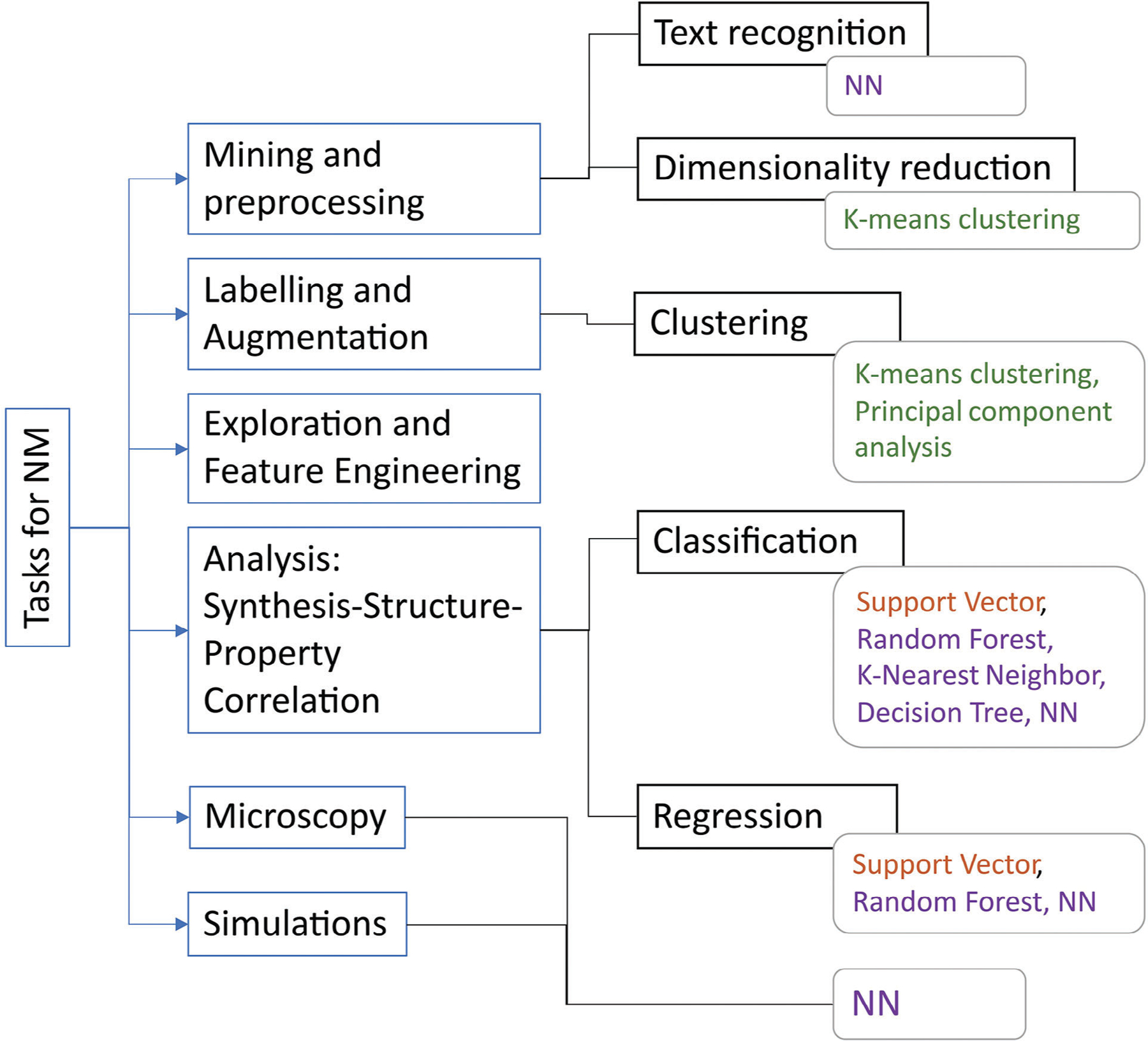
Summary of tasks and ML methods used in the investigation of nanomaterials. The types of ML methods are sectioned by color, with supervised methods being shown in purple, semi-supervised in orange, and unsupervised in green.

Some common tasks which ML can solve in nanomaterials research in particular, as well as the most used ML methods for these tasks are summarized in Figure 1, with common definitions and their associated abbreviations provided in the Supporting Information.

As a rule, the type of input data and the type of task determine which methods and ML algorithms can produce the best results. For example, for a classification task and data structured in a table, classical ML methods such as random forest (RF), K-nearest neighbor (KNN), and support vector machine (SVM) based on Kernel functions would be the optimal choice.^[17]^ In the processing of unstructured data such as images, spectral data, natural language text, and signals, the best results are achieved using DL. For example, computer vision techniques and DNN are efficient tools for image analysis including transmission electron microscopy (TEM), scanning electron microscopy (SEM), atomic force microscopy, as well as spectral two-dimensional (2D) maps.^[45–47]^ Moreover, for data mining of important aspects of NMs, such as their experimental design and properties, the texts of scientific articles can be analyzed using NN models optimized for language and natural language processing.^[48,49]^ Furthermore, spectral outputs such as absorption/emission intensity dependence on wavelength, including those with circular dichroism (CD), can be analyzed by neural models for time series forecasting and digital signal processing techniques.^[50]^ In the following section, we will provide some examples of ML methods for NMs and algorithms for solving some common research tasks.

In order to determine the optimal way to apply ML in your research, several steps should be considered, which are listed below.^[30,51]^

1. Define the research problem to be solved in your research area. How can this task be translated into terms of ML? Determine which class of ML *tasks T* your problem belongs to (classification, regression, clustering, anomaly detection, etc.).
2. Define a *performance measure P*. *Measure P* depends on *task T* and shows whether the ML model solves your original problem well. For example, this can be an accuracy metric or an F-measure (test’s accuracy) for classification tasks, and the values of *R*-squared (*R*^2^) or MRSE (Mean Root Square Error) for regression problems.
3. Collect and pre-process the raw data, forming a *dataset*. You may also need to label data, which is referred to as *data labeling*.
4. Explore the dataset with *Exploratory Data Analysis* where you understand the data in detail via data visualization and statistical analysis. This step provides a distribution of various *features* and evaluates the quality of raw data. Construct a matrix of feature correlations between themselves and the *target variable* that needs to be predicted.
5. Apply techniques such as *feature engineering*. This step involves performing data analysis, correcting inconsistencies in a dataset as incomplete, incorrect data or anomalies, scaling, normalization, and standardization. It is especially important for classical ML methods, such as RF, gradient boosting trees, logistic regression without NNs, and DL. After implementation of steps 1–4, a dataset should contain a description of the object *features X* and the *target variable Y*.
6. Select appropriate ML *model M* for further analysis.
7. Carry out *data processing* depending on the type of *selected model M*. Subsequently, split the dataset into a *training set* on which the model will be trained, and a *test set* that was not previously seen by the model for the final evaluation of the *measure P*.
8. Conduct *experiments* with the training of various *models M*, i.e., extracting *experience E* from the *training data*, and *evaluate* the results on a *test dataset* of how the performance of a model grows by *measure P*.
9. Try to *improve* the results. For example, you may use an ensemble of several strong models and/or take a large pre-trained NN model and fine-tune it on your data.
10. The trained *models M* make predictions (or *inferences*) on new data that the model has never seen before. Often, for this, the model is implemented as a *software service*.

For more detailed information, readers may refer to referenced books on the fundamentals of ML.^[32,33]^

## 3. Machine Learning Approaches in Nanotechnology

Nanotechnology is a rapidly growing area in materials science, a field in which massive amounts of data are produced via synthesis, research, optimization, and application testing of NMs. The discovery and optimization of novel materials can be facilitated by ML, since ML enables one to determine the relationship between synthetic parameters, structure, and properties of NMs using information from existing databases, reducing or eliminating the need for traditional time- and resource-consuming lab experiments and/or computational simulations. Data quality is a crucial factor in making accurate ML-based predictions. Thus, in this section, we place significant emphasis on the discussion of data quality and representation, coupled with the methods of data exploration. Many examples of ML methods are summarized in this section, as well as in Tables S1–S8 (Supporting Information).

For the researcher, it is very important to set the aim of the investigation (Step #1 “Define research problem” from Section 2) and what result is expected, i.e., in ML language, performance measure or accuracy of the model prediction (Step #2 from Section 2). Then, one needs to collect and pre-process the raw data as highlighted in Step #3 (Section 2), which is discussed in the next section in detail.

### 3.1. Nature of Nanomaterials Data

**Figure 2.**
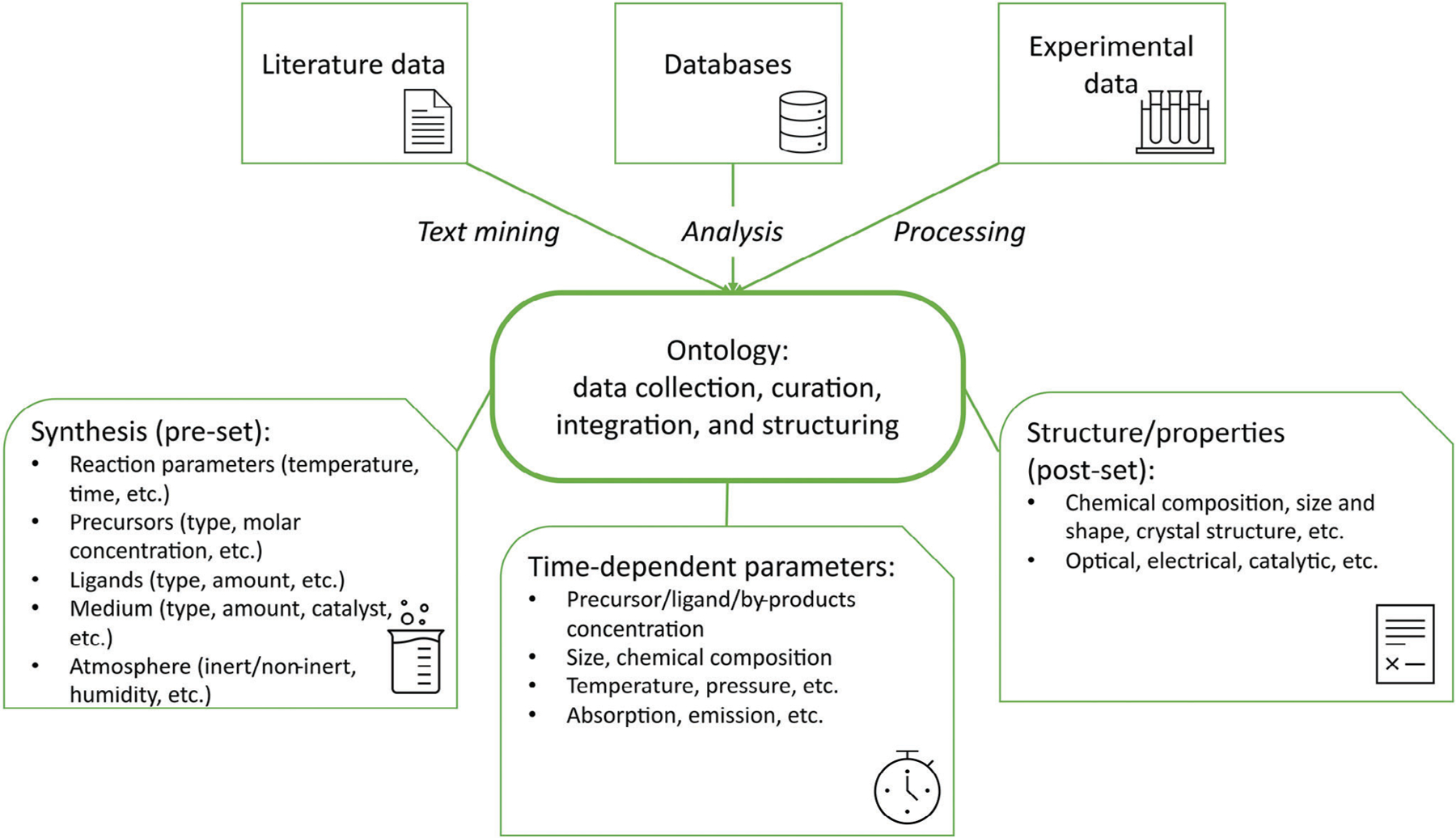
Data for nanomaterials: data sources, creating data ontology which consists of pre-set, time-dependent, and post-set parameters.

The diverse landscape of NMs is constantly expanding, which in turn results in the generation of enormous volumes of experimental data. Even within one synthetic methodology, the structure and properties of individual NPs might be slightly different. For example, the “wet chemistry” synthesis of NMs, including hot-injection,^[52]^ microwave-assisted,^[53]^ hydro/solvothermal,^[54]^ and pyrolysis,^[55]^ even with fixed reaction parameters, may result in the formation of NMs with slight structural or compositional differences, and hence changes to their properties and performance. To understand the intricate interplay between synthesis, structure, and properties, a parameter space should be constructed that will contain all possible data for further analysis. A parameter space can be described as the realm of possible parameter values that define a given NM. In this space, one typically can distinguish between two types of data: synthesis-related and property-related (Figure 2).

#### Synthesis-related data:

Synthetic parameters are most often defined by the researcher. To reveal the impact of a specific synthetic parameter on the resultant properties of NMs, a series of syntheses can be conducted, in which all synthetic parameters are held constant, except for one, which is systematically varied using a one-factor-at-a-time approach. This data is typically presented in the literature in the form of tables containing several parameters, such as the molar ratio of precursors, temperature, reaction time, etc., that are varied in a limited span, thus narrowing down the variables in the synthesis–property dependence. This approach is sophisticated and demands significant time and resources. Moreover, some of the parameters that influence the resulting NM during the synthesis, such as humidity, atmosphere (i.e., inert vs exposed to air), pressure, storage conditions, etc., are not consistently reported in protocols, imposing a certain degree of uncertainty on the inferred relationships (Figure 2, left panel).

#### Property-related data:

Another type of data is related to the properties of synthesized NMs, including morphology, and optical and electrical properties, among others. These properties are often evaluated via various characterization approaches, from visualization of the size and shape of nanomaterials using electron microscopy to the investigation of specific properties, for example, chiroptical responses (Figure 2, right panel). The collected data are often presented in the form of:

- numeric values (e.g., the energy of the highest occupied molecular orbital);
- *xy*-arrays (e.g., spectroscopic data);
- *xyz*-arrays (e.g., images or photoluminescence excitation-emission maps, to provide a few examples).

An additional type of data that can be collected is time-dependent data, for example, change in concentration of precursors and ligands, and the chemical composition of NMs as the reaction proceeds (Figure 2, central panel). This type of data is vital for shedding light on time-dependent processes that occur during the synthesis, formation, operation, and storage of NMs of interest.

##### 3.1.1. Challenges in Data Analysis

For data analysis, one typically uses software packages such as OriginLab, Wolfram Mathematica, MatLab, etc. However, manual data processing is often time-consuming. Furthermore, the occasional omission of errors and uncertainties from laboratory equipment and experimental setups, along with the constraints of finite data ranges, can limit the analysis and further implementation of derived dependencies.^[4]^

Furthermore, in order to build a sufficiently complex model for an accurate description of synthesis–structure–property correlations, it is insufficient to obtain data from one set of experiments, and it is of the utmost importance to collect as much data as possible. Tremendous amounts of data are dispersed across many different sources, including in-depth discussion of results in the literature, which can be extracted by text mining methods. However, it can often be difficult to collect and structure this data in a “machine-friendly” format, as was mentioned in Step #3 in Section 2.

##### 3.1.2. Databases

Usage of databases gives materials scientists access to enormous and diverse datasets, which are vital for accurate data analysis, simulations, and ML model training. Several examples of existing databases and their parameters are provided in Table S1 (Supporting Information). Many of these existing databases are focused on crystal structure, energy level structure, and physicochemical properties of bulk materials, while the NM databases are focused mainly on interaction with bio-objects, i.e., nanotoxicity. As an example, the NanoE-Tox database is based on existing literature on the ecotoxicology of eight NMs with different chemical compositions.^[56]^

The existing databases can be used as a starting point for further predictions and explorations of new materials with desired properties, as was recently demonstrated for ligands for A*β*(1–42) fibrils,^[57]^ and for 2D materials.^[58]^ Another facility that is particularly useful is Matminer—a Python library for data mining materials properties from online repositories and existing datasets.

These databases are constantly being replenished and enriched with new and updated data. The guiding principles for curating machine-readable data for NMs have been highlighted extensively in a recent article from Blekos et al., and underscore the importance of key considerations in the data collection process.^[59]^ These include prioritizing accuracy, completeness, and flexibility, to enable encoding of morphology and composite materials properties, distributions of properties, capture of auxiliary information, and reuse of data. The above-mentioned data sources and types of data are also summarized in Figure 2.

##### 3.1.3. Data Quality and the Data-Centric ML Approach

Even with the existing databases, data can often be disorganized, incomplete, and scarce. Data quality may depend on a wide range of factors, from physicochemical characterization requirements to broader issues such as minimum information checklists, and toxicology data quality schemes.^[60]^ The traditional strategy of improving ML models through a *model-centric approach* may not be the best way to enhance the performance of models and gain deeper insights into NM properties. Instead, the *data-centric AI approach* proposes to focus on improving data quality, rather than exclusively concentrating on model refinement.^[61]^ Thus, data quality is of utmost importance for further ML analysis and should be evaluated using the following criteria: accuracy, completeness, reliability, relevance, consistency, and timeliness, i.e., whether it’s up to date. Researchers in materials science are likely to face some of the following obstacles during the collection, pre-processing, and preliminary analysis of data which determine the *data quality*. Some indicators of poor-quality data include:

- Heterogeneous (data of different types, and measured using inconsistent scales)
- Incomplete (not all parameters or features are measured, there are gaps in the data space)
- Inaccurate (data is measured with errors)
- Inconsistent (while objects are the same, results are different)
- Insufficient (fewer examples are collected than features)
- Unstructured (no ready-made features present in the dataset)

Since maintaining high-quality data is a difficult and complex task, in a data-centric paradigm researchers should think about scientific data management and stewardship. To this end, the *FAIR* data principles have been developed, with the acronym representing the goal of Findability, Accessibility, Interoperability, and Reusability of data.^[62]^ These principles are widely supported by researchers and have inspired many *open data-sharing initiatives* in the realm of NMs.^[63]^ For example, Nature Nanotechnology recently announced their support of open data sharing inspired by the FAIR principles and strongly encourages their authors to do so.^[63]^ Furthermore, the open resource Nano Commons, which was established as a data and nano-informatics resource for the nano-safety community, offers an extensive guide for researchers in evaluating data completeness and data quality. Interested readers may also refer to a recent review from Yan et al. on how AI, in conjunction with molecular simulations, can enhance large-scale data analysis in nanotoxicology research.^[64]^

##### 3.1.4. Data Quality Assessment and Enhancement

It is important to draw attention to several examples of data quality assessment using ML approaches presented elsewhere. A comprehensive methodology for automated assessment of data quality and completeness, using an open-source R tool, is presented by Basei et al.^[65]^ In their approach, the authors present an approach for assessing the completeness of data by considering key materials properties (11 measured physicochemical properties) and testing procedures. From this, a completeness score was derived as the ratio of the number of key properties reported to the number of properties required by the database. In addition, the data relevance was assessed by classification of the data into 4 categories based on the toxicity studies protocols given in the data source and their automatic comparison with the pre-defined list of protocols. Furthermore, ML is likely to be incredibly useful in enhancing or improving the quality of existing data.^[66]^ A good example of this use of ML is provided by Walker et al., in which the data was initially converted to machine-readable format using RDKitpackage.^[67]^ The data quality was then further improved by firstly removing statistical outliers, followed by prediction error optimization using a NN. The importance of data quality and its open-accessibility and availability has been also discussed in detail elsewhere.^[68,69]^

##### 3.1.5. Data Mining Remarks

From the aforementioned obstacles, it is clear that data mining is a crucial but complicated aspect of ML. Addressing the challenges in relation to data quality requires strategies such as the synchronous use of multiple databases, and formatting data in machine-friendly formats. For example, the adaption of the chemistry-aware natural language processing tool (ChemDataExtractor) alongside the Cambridge Structural Database facilitated the building of an open-source database of metal-organic frameworks focused on their synthetic properties.^[70]^ Furthermore, this database also matches specific metal-organic frameworks (MOFs) to a list of known linkers provided by Tokyo Chemical Industry UK Ltd., which allows researchers to analyze the cost of these important chemicals. Increasing the speed of data analysis, i.e., queries and visualization processing, can be achieved by the arrangement of the data in a graph database, as was reported recently for properties of silica aerogels^[67]^ with Neo4j as a visualization tool, and for universal MatErials Graph Network (MEGNet)—molecules and crystals database.^[71]^ Using this MEGNet model in conjunction with density functional theory (DFT) calculations, Mao et al. were able to identify 3 promising electrets from unoptimized molecular conformations.^[72]^ The development of automatic evaluation methods of NM data quality and completeness is another means of shifting to the data-centric paradigm. A recent example of this from Basei et al. proposed an approach for the automatic assessment of NM data completeness and data quality for risk assessment purposes.^[65]^

To summarize, for ML approaches to help overcome the low quality of available data, large open-source datasets are required. Large amounts of data enable pre-treatment procedures to be applied, typically with the use of NNs—allowing for incomplete datapoints and outliers to be removed, resulting in a large amount of high-quality data. For further information on this topic, interested readers may refer to the paper by Batini et al. where the authors compared 13 methodologies of data quality assessment and derived a list of quality assessment steps.^[73]^ Another nice example of the influence of data quality on ML model accuracy is Chen et al., in which the authors compared different databases of medical entries, and showed that the accuracy of the ML predictions is highly dependent on whether the training database aligns with the specific task at hand.^[74]^ Furthermore, their study highlighted the significance of pre-training ML models on extensive datasets before applying them to the dataset of interest.

Aside from data quality, the volume of data required to build accurate and robust ML models is another vital parameter to consider. In a recent paper from Liu et al., the authors analyzed the fine balance needed between data quantity with ML model parameters and their accuracy for materials design and discovery.^[75]^ The authors highlight that, in materials science, due to the time and labor-intensive experimental data acquisition, the number of samples is often less than the number of features, leading to poor performance of the proposed ML models.

### 3.2. Data Labeling and Augmentation

In addition to the assessment and improvement of the quality of raw data, *data labeling* is another important step in data preparation for supervised and semi-supervised ML methods. *Data labeling* is the process of assigning labels to each of the objects for their further use.^[76]^ As a rule, relying on a single expert to assign labels to each object is insufficient. This is due to the possibility of human error, as well as discrepancies in approaches to labeling by different experts, which can be mitigated through cross-validation of results. However, labeling data with a high level of expert agreement is an expensive task. To simplify this process, various annotation services have been created, and a crowdsourcing approach is often applied for manual scientific data labeling.^[77]^ For the annotation of nanomedicine databases, Lewinski et al. asked experts to manually annotate drug labels, as well as training non-experts in the same task.^[78]^ The comparison of the annotation from the experts with that from non-experts showed overall good agreement, and, thus, can be further used for the labeling of the data. Methods for automatic labeling are also in active development. For example, Schwenker et al. developed a toolkit for the formation of a self-labeled database of electron microscopy images of NMs.^[79]^ Using their software, one can input a query, the data is collected from open-source articles, then each figure in the article is provided with a ML-generated figure caption, which serves as a data label. This is achieved via i) part-of-speech tagging, ii) division of a caption into small parts, and iii) formation of an individual caption for each part of the given figure. Next, all figures containing electron microscopy images are divided into individual images using object localization and object recognition tools NNs. The result is a self-labeled database of microscopy images with individual descriptions and captions.

In addition to labeling substantial volumes of data, an alternative approach, known as data augmentation, is often used for training NNs to expand the training dataset. Data augmentation is a technique that involves artificially expanding the training dataset by generating modified copies of an existing dataset. An example of this is described by Luo et al., in which data augmentation techniques and a transfer learning approach were used for improved DL classification of TEM images of carbon NMs.^[80]^

### 3.3. Data Exploration with Supervised Learning

At Step #4 mentioned previously, i.e., exploratory data analysis, one needs to “understand” the collected data. In this regard, one can determine the following values for each parameter: mean, standard deviation, minimum and maximum, Q1–Q3 interval, median, and outliers. The target variable or parameter of interest, i.e., NP size to give just one example, is in most cases estimated poorly on large and scarce datasets. For better accuracy in the estimation of a parameter of interest, it is necessary to divide the data into groups and establish the key features which most influence the target variable or parameter of interest. An example of the power of ML in identifying the correlation between NMs properties was reported recently by Suzuki et al., in which the authors were able to decode the relationship between DFT-calculated electron affinity and ionization potential values and electret performance via Pearson correlation.^[72]^

Further data exploration requires splitting parameters of interest into smaller groups, which increases the accuracy of further analysis within a class. Dividing the data into the groups is a classification task, that can be solved manually, or by using supervised ML methods such as SVM, RF, KNN, decision tree (DT), and NN.^[81]^ For example, supervised ML classification based on classifiers with linear discriminant analysis (LDA) demonstrated the discrimination between natural and engineered silica NPs with an accuracy up to 93.3%.^[82]^ Classification can be also carried out using DT, RF, radial basis function SVM, and linear SVM classifiers, as recently demonstrated by Ma et al. to divide polymer nanocomposites based on the glass transition temperature.^[83]^ The RF and DT classifiers demonstrated classification accuracies of 0.922 and 0.852, respectively, on a dataset of 120 nanocomposite samples. NNs are also commonly used for classification tasks. A recent example of NN-based classification for NMs from Bang et al. was capable of predicting the adsorption energies of NPs by taking into account different bonding types, to understand their stability in electrochemical environments.^[84]^

Data exploration step is then followed by Step #5, feature engineering, to reveal the correlation between parameters and target variables for further analysis and modeling with classical ML methods. Identifying the correlation can be done in numerous ways, including via correlation matrices, SHapley Additive exPlanation (SHAP), missing data imputation, categorical encoding, feature transformations, and splitting, discretization, and outlier engineering. For example, SHAP can be used as a part of a motif extractor algorithm to identify the importance of each feature for further target prediction. This was demonstrated by Anker et al. using an example of the effect that the presence of individual atoms in a C_60_ buckyball has on the quality of the fitting X-ray PDF data.^[85]^ Similarly, SHAP was applied by Ma et al., to identify 10 nano-descriptors of functionalized gold NPs correlating significantly with dendritic cell activation, derived from feature importance calculations using a RF algorithm, for applications as adjuvants in vaccines.^[86]^ It is worth mentioning that the matminer open-source library, previously mentioned in this review, also has a “featurizer” tool, in which features of materials can be identified and derived from the available data.

### 3.4. ML-Enhanced Data Analysis: Identification of Synthesis–Structure–Property Correlations

Perhaps the primary use of ML to date in the study of NMs has been centered around the prediction of the structure and properties of substances based on the available experimental data. ML algorithms possess the ability to find connections between synthetic parameters and the resultant structure of materials. ML can also establish connections between various material properties, i.e., optical response and cytotoxicity, and the material’s structure and composition. By analyzing synthesis–structure–property correlations, ML facilitates the prediction of the properties of a given material using known synthesis parameters (forward prediction), and vice versa, to design optimal synthesis conditions for obtaining a material with desired properties (inverse design). Often, the same ML algorithm can perform both forward and inverse processes. Therefore, ML has the potential to significantly reduce and perhaps in some instances eliminate the need for laborious and time-consuming experiments and computer modeling. In essence, the in-depth analysis of the synthesis–structure–property relationship allows for the optimization of synthesis to achieve desired properties, along with the prediction of material performance.

As soon as one sets *target variable Y* and *features X*, the next step is the selection of an appropriate ML model according to the defined problem, as mentioned in Step #6 previously in Section 2. Further analysis of the data is then conducted according to Steps #7–9. Several different ML models and algorithms or their combinations can be applied and compared to achieve higher accuracy. For example, four different ML algorithms, i.e., DT, KNN, adaptive boosting (AdaBoost), and RF were used to facilitate the optimization of Fe–Ni–Ti–Al maraging steel using data on phase contents and critical temperatures.^[87]^ In the following subsections, several examples of the application of ML in the data analysis of NMs are discussed.

#### 3.4.1. Synthesis–Structure–Properties Correlations

Synthesis–structure–property relationships in materials can be decoded and revealed using ML regression models. Those models include multipower regression, RF, K-nearest neighbor, support vector (SV), and principal component analysis (PCA) together with NN, etc.^[83,87–89]^ As an example, Chen et al. employed a RF model to quantitatively analyze the Cu level in carbon black particles.^[90]^ The authors used laser-induced breakdown spectra which were used in modeling with and without pre-treatment with variable selection methods. It was demonstrated that optimizing the spectra prior to analysis can improve the accuracy of quantitative Cu detection. Another example of ML-aided spectral analysis is provided by Timoshenko et al.^[91]^ In their work, X-ray absorption near-edge structure spectra were firstly analyzed with PCA, one of the most commonly used unsupervised ML algorithms, to deconvolute them into specific components responsible for chemical composition—i.e., what determines the Co to Fe ratio, as well as phase transitions, in CoxFe3–xO4 catalysts. Subsequent to PCA, the authors developed a NN which allowed them to obtain vital information on the phases and oxidation states of the Co and Fe in the catalyst.

Another crucial task in the field of materials research is to establish the dependencies of the electronic structures of materials on their atomic structures, a task in which ML methods can play a huge role. In recent work from Alessandri et al., electronic properties of soft materials were connected to conformational degrees of freedom using a supervised NN model which was trained on coarse-grained representations of polymers.^[92]^ This approach was validated by a good agreement with results from DFT and molecular dynamics (MD) calculations, as well as enabling high-throughput experimentation with lower computational demand.

To improve the quality of approximation and prediction of NMs data, a combination of ML approaches can be used. Vizozo et al. aimed to find the connection between the vibrational density of states and deformation and defect states present in materials.^[93]^ In their approach, the authors firstly pre-processed the data from DFT and MD measurements by unsupervised learning for data dimensionality reduction using PCA, an approach mentioned in Step #3 of our guidelines above in Section 2. Next, they built a gradient boosting DT model that was capable of linking vibrational spectra to a variety of structural configurations.

Occasionally, analysis of spectral and structural data independently by different ML methods such as PCA and standard NNs may result in poor quality outcomes, as has been recently reported by Yaman et al.^[47]^ To overcome this problem, the authors developed a dual variational autoencoder to establish the correlation between SEM images and scattering spectra of gold NPs. This method allows for the analysis and prediction of geometries of plasmonic particles and their clusters via encoding the shape-spectra data space.

Analysis of datasets with ML also enables the discovery of complex relationships between several properties of a given material. For example, a multiscale structure–property correlation is reported by Liu et al. where experimental data on morphology including TEM/SEM images and X-ray diffraction (XRD) patterns, together with DFT results on energy structure were analyzed, followed by the application of graph convolution NN and LightGBM to the dataset.^[94]^ This approach allows one to predict the surface parameters of hydroxyapatite NPs, such as energy and stress, via explainable ML methods. Aoki et al. describe a similar analytical approach for polymer-solvent systems, in which they developed a predictive DL model which allowed for the pre-determination of features such as the classification of polymer miscibility along with the evaluation of thermodynamic parameters.^[95]^

#### 3.4.2. Prediction and Optimization of NP Properties

Several ML approaches can be applied for optimization and prediction of the properties of NPs properties, including various types of regression: NN, RF, DT, etc. For example, in work by Wang et al., data collected from syntheses of cellulose NCs with a focus on their chemical yield and crystallinity, was analyzed using DT, RF, and NN algorithms. The authors found that the RF algorithm was optimal for the prediction of the chemical yield, whereas a DT regression model provided the highest accuracy for crystallinity prediction.^[96]^ In other work from Hou et al., DNNs and an active learning (AL) scheme were used to develop a NN potential enabling the analysis of oxidation kinetics in MXenes, with the advantages of ab initio precision at lower computational cost. The NN potential was based on the representation of the energy of NP as a sum of the energies of each atom within the NP.^[97]^

Recent work from Kløve et al. described an “on-the-fly” trained ML-enhanced global optimization algorithm to predict crystal structures of TiO_2_ and SnS_2_.^[98]^ The proposed model was based on experimental pair distribution functions from X-ray and/or neutron diffraction total scattering data combined with DFT calculations. The proposed algorithm of energy optimization of the NMs via changes in crystal structure even allows for the identification of defects such as stacking disorder in materials, as well as metastable configurations, which could prove to be incredibly useful for the analysis of NMs with chiral defects. Wang et al. also reported the combination of laser-induced breakdown spectroscopy and support vector classifiers and regressors, to predict catalyst properties and serve as a guide to catalyst synthesis via flame spray pyrolysis. They demonstrated that crystal structure parameters of Ce-Zr-Mn catalysts could be predicted by labeling the laser-induced breakdown spectra with experimentally determined phase content, lattice constant, and oxygen vacancy with further use of ML methods.^[99]^

A combination of ML approaches can be also used for surface topology prediction. For example, He et al. used descriptors based on nanoscale roughness and microscale topographies in addition to the surface hydrophobic modification to offer a feasible solution to complex topographies.^[100]^ Further applied SHAP analysis allows for the extraction of water drop adhesion trends on superhydrophobic surfaces. Another example of the prediction of NP composition from Chen et al. is based on a combination of a mixed variable importance method allowing pre-treatment of the data, and RF calibration model, where the mean relative error of prediction decreased by 62% compared to RF-only model.^[90]^

In addition to composition-based predictions, NNs can be also applied for the prediction of NM properties. For example, a supervised NN was demonstrated to produce high-accuracy predictions of the optimal synthetic parameters for silica aerogels.^[67]^ A graph convolutional NN was used by Bang et al. in the prediction of electrochemical stability based on the crystal bond type. The developed NN allowed the production of reliable Pourbaix diagrams valid for large-size NPs involving up to 6525 atoms, or 4.8 nm in diameter.^[84]^

For the forward prediction of NM properties, two main classes of methods can be highlighted: evolutionary methods (genetic algorithm or particle swarm) and local techniques (adjoint method). The genetic algorithm can be utilized with a small number of parameters and on datasets lacking in large amounts of information. Genetic algorithms are inspired by natural selection and evolution, as described by Koza in his work.^[101]^ In this context, each individual in the algorithm represents a set of parameters (chemical composition, structure, etc.) which are considered as “genes.” The next generation is constructed by both a random modification of genes and a “crossbreeding” between two configurations, resulting in continuous improvement and optimization of the parameters. For example, the strong coupling between plasmonic nanocavities and monolayer semiconductors was optimized by a genetic algorithm with a novel figure-of-merit, with the evaluation of each configuration being based on the finite-difference time-domain method.^[102]^ Furthermore, genetic algorithms can be accelerated further by ML, as was shown for the discovery of stable, compositionally variant, geometrically similar NP alloys for catalysis, resulting in a 50-fold reduction in the number of required energy calculations compared to a traditional “brute force” genetic algorithm.^[103]^

#### 3.4.3. Closed-Loop Approach

**Figure 3.**
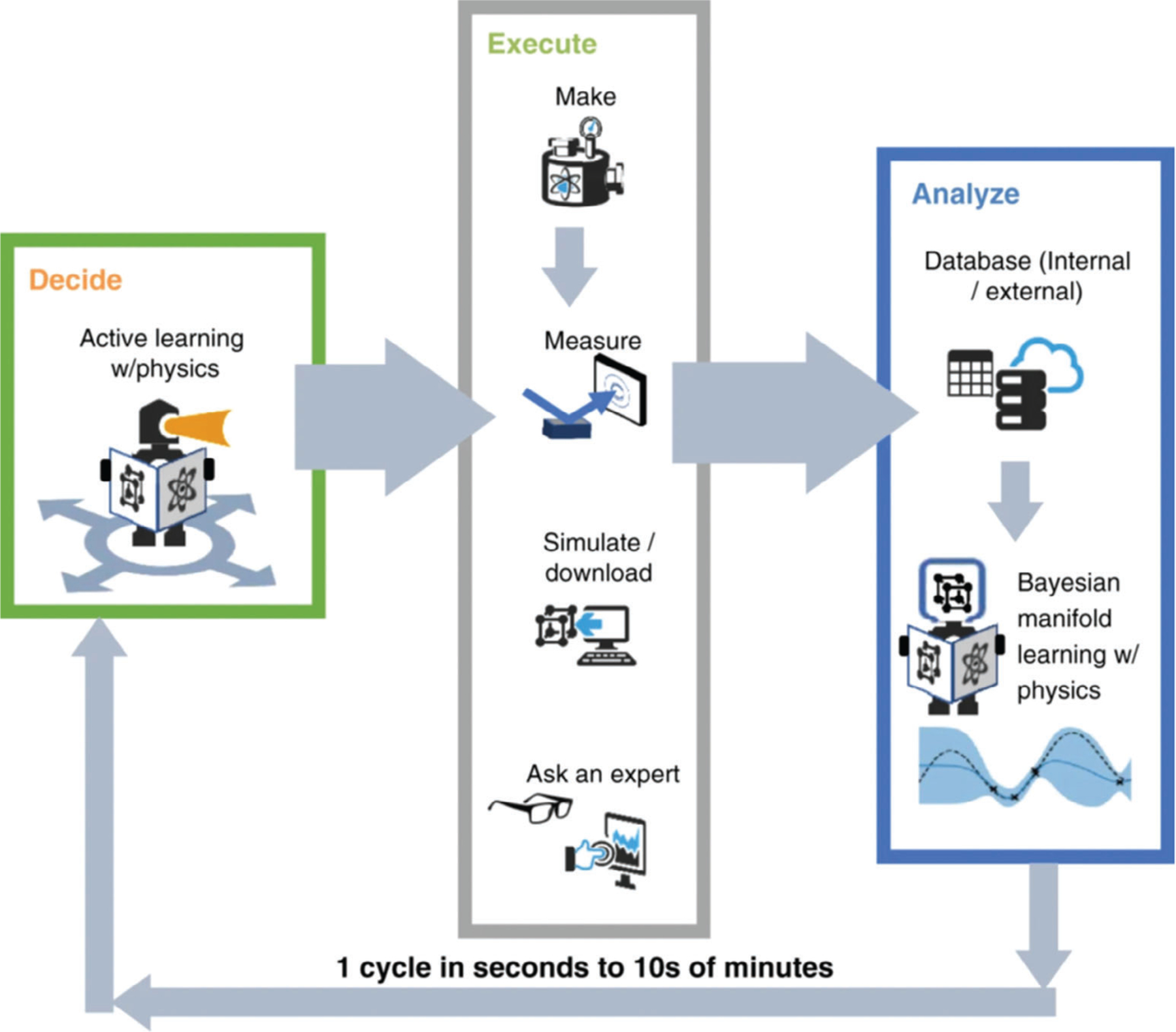
Schematic of closed-loop materials discovery via Bayesian active learning. Reproduced with permission.^[105]^ Copyright 2020, Nature.

Another approach that can facilitate the discovery of new NMs is active ML, which can be considered as a closed-loop “Learn–Design–Build–Test” process, as demonstrated by Tamasi et al. for polymer-protein hybrids.^[104]^ According to theirs, and other cited work,[87] this process of discovery and optimization of novel materials includes several stages, as illustrated in Figure 3:

- Initialization with a seed dataset and feature selection, followed by setting the limits to feature values (for example, concentrations of precursors, chemical composition, etc.).
- Learn: make predictions of specific parameters of the NM based on its characteristics by training Gaussian process regression surrogate model, DT, KNN, RF, AdaBoost, etc.
- Design: Bayesian optimization or a population-based meta-heuristic algorithm, i.e., Differential Evolution, can be used for NM to satisfy an expected improvement acquisition function and subsequent filtering to propose new materials.
- Build: automated synthesis of proposed NMs.
- Test: evaluation of properties of interest for selected NMs. The newly acquired and existing data are then used to begin a new Learn–Design–Build–Test iteration.
- Final decision.

The closed-loop approach can be also applied to the discovery of new materials with improved performance via error correction learning,^[106]^ which enables the model to learn from prior datasets, and then adapt to differences in synthesis and characterization that are otherwise difficult to parameterize. This approach can be implemented not only for properties of the NMs, but can also be applied to their assemblies, as was shown by Reker et al.^[107]^

#### 3.4.4. High-Throughput Experimentation with Microfuidic Reactors

For the discovery and optimization of synthesis–structure–property relationships in NMs, large amounts of reliable data are needed, which is often beyond the capabilities of what one laboratory may produce during a reasonable period. We previously discussed the use of existing databases and incorporating novel results into the existing data space, however, this approach possesses several disadvantages (discussed in “3.1. Nature of Nanomaterials Data”). Another way to generate a large amount of experimental data is using high-throughput microfluidic reactors.^[108]^ Microfluidic automated synthesis systems involve reaction chambers with precisely controlled conditions (temperature, pressure, etc.), through which precursors are streamed at a certain rate, and at the output, the reaction products are analyzed by various methods.^[9]^ One can automatically control the injection of precursors (one or multi-step, molar concentrations, temperature, etc.), flow velocity, and, hence, reaction kinetics, temperature, and pressure in each point of the microreactor, together with simple monitoring of products formed during reaction/flow, including absorption/emission spectroscopy.^[109]^ This enables researchers to acquire large amounts of data in a short time. An excellent review from Munyebvu et al. focuses on the use of high-throughput microfluidic synthesis in combination with ML to study the properties of colloidal quantum dots (QDs) and to facilitate the translation of QDs to production and applications.^[4]^ These relatively low-cost and simple systems can be designed to be extremely versatile and modular, can be easily customized from smaller components, and can be tailored to specific applications. Moreover, the data generated is standardized and already in a machine-friendly format, and several parameters can be collected/monitored simultaneously and continuously in time allowing the design of a high-quality data space efficiently.

In *closed-loop* formats, a mathematical algorithm can be used either to optimize the reaction for better performance of the NMs or to explore unknown synthesis–structure–properties space. For example, exploration, discovery, and optimization of seed-mediated multistep synthesis of gold NPs can be performed by an autonomous chemical synthesis robot.^[110]^ Using MAP-Elites and global search with local sparseness algorithms for NM exploration and optimization, respectively, on real-time spectroscopic feedback allows for the exploration of reproducible novel synthetic methods. Another example is reinforcement learning (RL) that enables the discovery and optimization of shell-growth for core-shell semiconductor NPs based on datasets of spectral characteristics, using self-driven fluidic lab AlphaFlow.^[111]^

### 3.5. Machine Learning-Assisted Microscopy

Detailed information on NM structure can often be acquired through microscopy imaging. ML is widely applied nowadays in this area. Regardless of the imaging approach (electronic, optical, or scanning probe microscopy), ML is commonly applied for two main tasks: i) image restoration to improve image quality; and ii) image recognition and classification.

#### 3.5.1. Image Restoration

ML presents new opportunities to solve classical problems in imaging, such as de-blurring, de-noising, and achieving super-resolution. There are several useful reviews on using ML for image reconstruction and enhancement in optical^[112,113]^ and electron microscopy,^[114]^ along with image de-noising.^[115]^

The current front-runner in ML-assisted image analysis is the convolutional neural network (CNN). In the classical approach, one uses sets of pairs of images pairs with good/bad quality to train a NN, effectively working as an informational black box with hidden or unknown rules for image restoration. In cases where it is not possible to produce the training set experimentally (i.e., record the same image with good and bad quality) ones often simulate images to mimic the appearance of real samples.^[114,116]^ For the task of training a NN for image analysis, one can also use images from online collections and databases of both experimental and simulated images of NPs, as well as atomically resolved material surfaces. A few imaging datasets are worth mentioning here: CNR—IOM SEM dataset,^[117]^ DeCost—Holm SEM dataset,^[118]^ TEM ImageNet, Warwick EM dataset,^[119]^ and Zelinsky Institute of Organic Chemistry SEM dataset.^[120]^

For routine denoising tasks there are two key approaches which enables the restoration of images in the absence of clean data, namely the Noise2Noise^[121]^ and Noise2Void^[122]^ approaches. The latter example does not require noisy image pairs in the training of a NN to produce denoised results. Both methods are based on the concept that target signal and noise have different spatial distributions, which allows one to train a NN to produce denoised images. For theoretical background of both methods, we refer readers to the original papers (mentioned above), while for the practical application of this ML-powered denoising approach, we encourage readers to visit the CSBDeep website.

**Figure 4.**
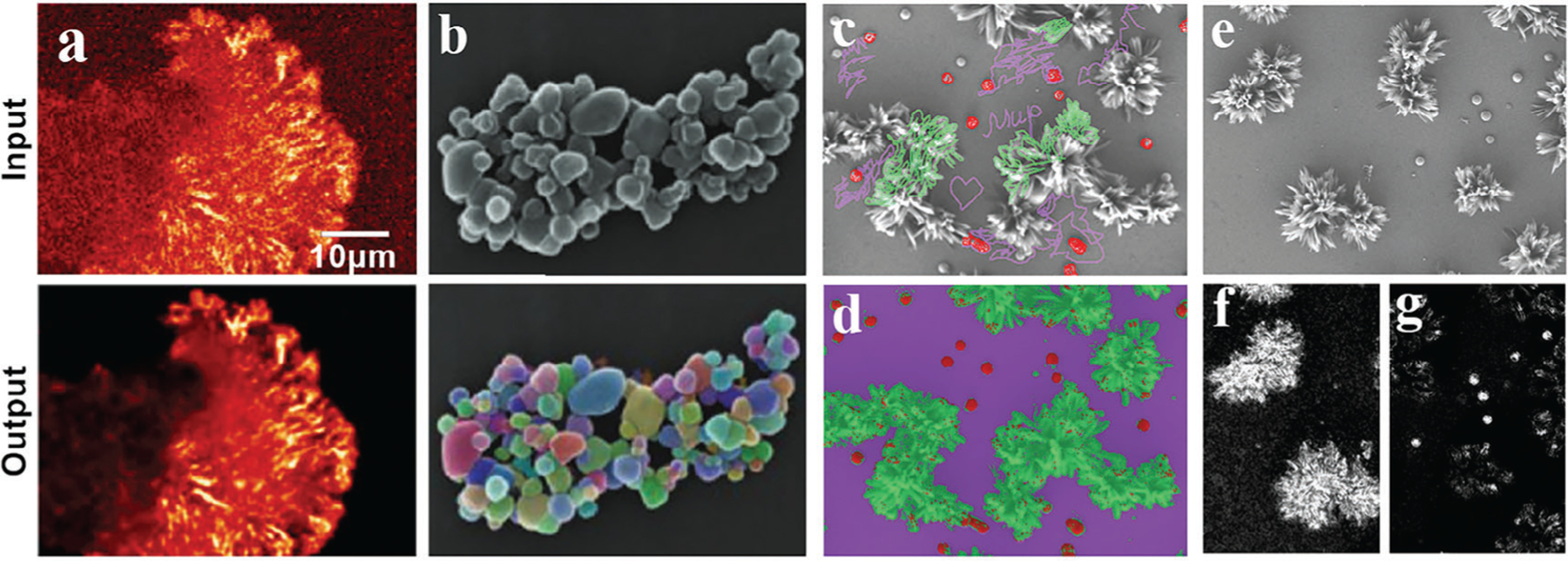
Images before (top panel) and after (bottom panel) ML-based treatment. a) Image of actin label (paxillin-GFP) in U-251-glioma cells denoised with using Noise2Void network. Adapted with permission.^[124]^ Copyright 2021, Nature; b) example of segmentation TiO_2_ nanoparticle image with using U-Net CNN. Adapted with permission.^[125]^ Copyright 2021, Wiley-VCH; Example of image segmentation with using Weka plugin:^[126]^ c) SEM image of self-assembled structures made of CdZnSeS/ZnS quantum dots with manual labeling of spherical (red), flower-like (green) structures and background (lilac); d) Weka segmentation output; e) SEM image that the classifier has not seen before; f,g) the probability maps of recognition flower-like and spherical structures, correspondingly. Adapted with permission.^[127]^

CSBDeep is an online deep learning toolbox for microscopists, which offers a collection of state-of-the-art methods for content-aware image restoration and segmentation. The website showcases several real-world examples with detailed instructions on how to apply the methods to a researcher’s own data treatment. Figure 4 presents an example of image denoising, which can be done using the Noise2Void approach, according to the detailed instructions presented on CSBDeep website.

Despite the significant strides the aforementioned techniques have made in image quality improvement, it seems like the true future of this area lies in the vision transformer (VT) architecture.^[38,123]^ VT has numerous advantages over current approaches: such as improved efficiency (especially when more data is fed to the network), robustness in feature extraction, and a better feature learning approach that can more accurately capture the variances and characteristics of the input. Nevertheless, some drawbacks exist, such as the need for more data to show the benefits of VT over DNN, the increased computational cost due to the complexity of the self-attention block, a more challenging training process, and the lack of interpretability. However, considering the rate of data generation and pace of AI development, there is little need for concern over these limitations. It is possible that, in the near future, every imaging research software could feature a simple “image enhancement” button.

#### 3.5.2. Image Segmentation and Classifcation

The progress in microscopy techniques and the rapid growth in the volume of generated imaging data underscores the need for automated image analysis. Prior to ML-driven analysis, structures of interest must be identified and represented in machine readable formats. This can be achieved through segmentation, a process of converting an image into regions or segments that are represented by a mask or a labeled image (Figure 4b). Another similar operation is image classification—the process of categorizing and labeling groups of pixels or vectors within an image based on specific rules.

Nowadays, the most common ML approach to image analysis is the CNN, and the most popular architectures for image segmentation are SegNet, U-Net, and Mask R-CNN. A recent article by Cheng et al. compares the classification networks commonly used in materials science, demonstrating that ResNet18 has a lower computational cost and better performance among other CNN architectures.^[128]^ Most notably, ResNet18 was shown to work even better than the VT approach, which is the state-of-the-art network for the manipulation of natural images (images that a human being would observe in the real world). To date, we have found only a handful of papers in the area of NMs that use vision transformers for image recognition. Transformers were used for accurate measurement of NP size from TEM images,^[129]^ as well as for automated registration of electron microscopy^[130]^ and medical images.^[131]^ However, we envision that the performance and usability of transformers will experience rapid and substantial growth as further research is conducted in this area.

For those who are searching for a quick practical solution for ML-assisted image analysis, we can recommend an open-source ImageJ plugin called Trainable Weka Segmentation (TWS).^[126]^ TWS is a ML tool that demands a limited number of manual annotations in order to train a classifier and segment the remaining data automatically, as demonstrated in Figure 4c–g above. In addition, TWS can provide unsupervised segmentation learning schemes (clustering) and can be customized to employ user-designed image features or classifiers. AtomAI is another open-source software package which facilitates the direct application of DNNs for atomic and mesoscopic image segmentation, which is particularly useful for HRTEM and STEM data analysis.^[132]^

### 3.6. Machine Learning for Simulations: Make It Faster, Make It Better

In the modern era of materials science research, computational simulations are a powerful partner to experimental studies. Simulation facilitates a better understanding of the properties of existing materials, enabling the determination of the close relation between their structure and properties. Additionally, optimization through simulation can lead to the discovery of new, improved materials with enhanced performance. In the realm of simulations, NMs can be considered on either the electronic or molecular level.

The electronic level is simulated by DFT, a methodology based on quantum mechanics. This approach is usually applied for the calculation of the electronic structure of atoms, molecules, and solids.^[133]^ The DFT method typically gives the most precise results, however, because of high computational cost, it is generally restricted to systems with fewer than 1000 atoms.^[134]^ Moreover, even though a universal exchange–correlation functional, which describes the electron density, theoretically exists, the explicit form of the functional is unknown.^[135]^

The investigation of the molecular or atomic level is conducted by MD or/and Monte Carlo simulations. MD simulations use the laws of motion, energy, and forces to model interactions between all atoms in a system. Compared to DFT, MD is a computationally inexpensive approach, however, its accuracy is entirely dependent on empirical interatomic potentials. These empirical potentials are available in very limited quantities, especially for multi-component systems. Monte Carlo simulations use random sampling methods to generate different molecular configurations, facilitating the discovery of the optimal state, and calculation of the thermodynamic properties of a system. Unlike DFT and MD, Monte Carlo simulations have no physical nature and do not provide any information about time evolution. Nowadays, MD and Monte Carlo approaches are most often used in conjunction with one another.^[136,137]^

The primary objective of the application of ML in NM simulations is the reduction of computational cost, while simultaneously increasing accuracy. For interested readers, there are several reviews published on the application of ML in computational nanotechnology, and in DFT,^[138]^ and MD,^[139]^ specifically.

The most radical approach to minimize computational costs is to avoid simulations at all, instead opting for training NNs to undertake the entirety of a task. In this case, data obtained by experiments and previous simulations can be used to train and validate a NN. Such an approach allows one to extend the prediction of electronic structures to much larger length scales, overcoming a key limitation of DFT.^[140]^

Combining ML and DFT calculations enables a systematic investigation of the intrinsic connection between the structure and properties of potential materials. The DFT-ML framework screens structures with desirable target values by the established ML models and subsequently validates these materials via DFT calculations.^[141,142]^

ML also can be harnessed for finding improved functionals and interatomic potentials for DFT and MD calculations, respectively.^[143]^ This approach is an extremely popular direction of work since precise interatomic potentials can theoretically increase the accuracy of MD to the level of DFT, with much more moderate computational cost. Application of NN potentials, termed “deep potentials”^[144]^ enabled researchers to perform simulations of 100 million atoms with ab initio accuracy.^[145]^

In summary, the advancement of ML-assisted computer simulations is propelling rapidly toward the capability of the prediction of properties of any possible material with near ab initio precision, in increasingly shorter times^[146]^ and at increasingly massive length scales.^[140]^

### 3.7. Ready-to-Use ML-Based Software Packages for Non-Specialists

To date, there are several ML-based software packages aimed at solving a multitude of problems in materials science, including the design of novel materials with desired properties, that allow even users without minimal coding experience to use ready-made packages for the analysis of NMs. AlphaMat is one such package, that can be used to design materials with desired properties or to predict properties of existing materials, such as formation energy, band gap, ionic conductivity, magnetism, phonon property, bulk modulus, dielectric constant, adsorption energy, etc.^[147]^

Moreover, the interaction between nano- and bio-objects can be predicted using the ML pipeline developed by Saldinger et al., based on the coarse-graining method to generate a rototranslational equivariant representation of NPs, and DNN that predicts pairwise interactions between the coarse-grained sites of two objects.^[148]^

Another example of AI and ML application in the NM field is in microscopy image processing and analysis, as has been discussed previously in detail. The AtomAI framework is an example of a user-friendly package which can utilize DNNs for atomic and mesoscopic image segmentation, converting images and spectroscopy data into class-based local descriptors for down-stream tasks.^[132]^ In addition, the program facilitates the analysis of spectroscopic data and the identification of structure–property relationships. Moreover, virtually all commercially available microscopy image analysis software nowadays routinely incorporate AI technology in their products. Aivia (Leica), Amira-Avizo (Thermo Fisher Scientific), Imaris (Oxford Instruments), and ZEN (Zeiss), to name a few, all contain predefined workflows for image segmentation, object classification, counting, and tracking.

Another useful option for ML implementation in image analysis is DeepImageJ: a user-friendly e-plugin that enables the use of a variety of pre-trained NNs in ImageJ/Fiji open-source software.^[149,150]^ The plugin is actively supported on GitHub, so anyone may contact the community for any questions.

## 4. From Predictive Power to Automated Analysis: The Role of Machine Learning in the Promotion of Functional Nanomaterial Applications

By leveraging its large-scale analytical capabilities, ML has paved the way for further exciting opportunities in the discovery and advancement of the wide-ranging applications of functional NMs. The unparalleled ability of ML to analyze and generate massive datasets, as well as identify and predict patterns and trends, is helping researchers uncover unique properties and thus the suitability of NMs for tailored applications. Furthermore, it is envisioned that ML has the potential to predict multifunctionality in existing materials, reducing our reliance on traditional, time-intensive experimentation.^[151]^ This ML-driven acceleration of NM applications is expected to lead to significant breakthroughs in medicine, advanced separation technology, catalysis, and electronic devices, among others. While the primary focus of this section is on utilizing ML to predict material performance, as well as aiding in the analysis of complex data, it is important to recognize the intrinsic link between material properties and their applications, as has been highlighted throughout this review thus far. As such, this section sheds light on the pivotal role that machine learning can play in the implementation of functional nanomaterials in real-world applications.

### 4.1. Filtration and Separation Technologies

ML has the potential to play a pivotal role in expanding the horizons of advanced filtration and separation technologies. ML algorithms can be developed and applied for optimization of materials and membrane pore sizes, tuning of selectivity and permeability, as well as understanding mechanisms and competing separation processes.^[152]^ ML is increasingly being utilized in the assessment of gas separation membrane performance according to several recent articles.^[153–155]^ However, perhaps the most studied NM-based separation application using ML in recent times is filtration—namely ultrafiltration, nanofiltration, and reverse osmosis.

Fetanat et al. built a database through the compilation of 14 years of published nanocomposite ultrafiltration membrane data.^[156]^ A total of 2300 NN models were trained and tested using this data to predict key performance indicators for ultrafiltration membranes. The top 30 models, which were found to predict solute rejection and water flux with high accuracy, were used in the building of an open-source program with a graphical user interface based on MATLAB. This program enabled materials scientists with no prior coding expertise to predict performance for new ultrafiltration membranes using input variables such as solvent type, particle size, membrane materials, and solvent contact angle. Wang et al. conducted a study extracting data from 20 papers to create ML models for the prediction of thin-film nanocomposite membrane performance for organic solvent nanofiltration.^[157]^ A gradient-boosted tree model was found to outperform other models in terms of the prediction of permeability and selectivity of the membranes. Additionally, this model was utilized to identify the key parameters influencing permeability and selectivity. Yeo et al. used a gradient-boosting tree model to assess the impact of key parameters such as pore size, NP size and loading on thin-film nanocomposite reverse osmosis membranes.^[158]^ Their study revealed that factors such as NP loading, shape, size, and pore size have a crucial influence on water flux and salt rejection in reverse osmosis membranes, offering valuable design guidelines for future membrane development.

However, it is important to highlight that the application of ML in filtration and separation technologies is still in the early stages of development, due in part to incompleteness of data, inappropriate data treatment, and data leakage. A study conducted by Jeong et al. using the XGBoost ML algorithm demonstrated that, while ML can be applied for the prediction of the size-exclusion performance of reverse osmosis and nanofiltration membranes, the model is limited in its understanding of adsorption processes and electrostatic interactions.^[159]^ Therefore, it is evident that more high-quality and diverse datasets are required to improve the training and thus the predictive capability of ML models, in turn aiding in the advancement of the application of ML for separation technologies. Nevertheless, the close relationship between ML and experimental materials research presents significant opportunities for separation science. As ML is already being utilized in the field of chiral separation in the form of chromatography, it is envisioned that the advancement of ML should also uncover new opportunities for chiral NMs, such as ultrafiltration and nanofiltration membranes for chiral separation.^[160–163]^

### 4.2. Sensing

ML is increasingly proving itself to be a game-changer in the field of NM-based sensors, enhancing selectivity, sensitivity, and analysis, as well as the discovery of new NMs for sensing. ML-assisted NM-based sensors are enabling more accurate and efficient sensing across a diverse range of applications, from environmental monitoring to disease diagnosis.^[164,165]^

Wan et al. used ML for modeling potential sensors for detection of the gaseous decomposition products of CF_3_SO_2_F—an eco-friendly gaseous electrical insulation medium.^[166]^ The authors performed a high-throughput screening study of a large variety of transition metal-embedded graphitic carbon nitride, with the primary objective being the ML-powered prediction of the interaction strength between the transition metal-embedded graphitic carbon nitride and the gaseous decomposition products. A hybrid DFT/ML method was applied, using 8 supervised ML algorithms, with support vector regression being identified as the optimum model. The approach identified 4 new sensing materials for CF_4_, HF, SO_2_, and SO_2_F_2_ gases. Singh et al. used ML to aid in the understanding of the performance of their developed MoS_2_/carbon nanotube (CNT)-based sensor for the detection of DMF and NH3, with applications in air quality monitoring and disease diagnosis.^[167]^ A python-based ML model was used to conduct PCA by feeding the experimentally obtained gas responses into the algorithm, resulting in variance outputs which could be attributed to primarily two principle components. The PCA served as evidence that both the sensor response and the recovery time were highly dependent on the gas concentration and also confirmed the sensitivity of the nanocomposite-based sensor toward NH_3_ and DMF detection in the presence of other gases. Huang et al. developed a new ML model to support a nanofiber-based colorimetric sensor for environmental Cr(VI) monitoring.^[168]^ The authors developed a novel few-shot learning ML model, based on a combination of a deep CNN and a generative adversarial network, to extract color features from smartphone images of the colorimetric sensor, leading to an increase in accuracy from 51% to 85%. Furthermore, the implementation of the few-shot learning improved the Cr(VI) detection limit from 1.571 to 0.05 mg L^−1^, and allowed for accurate Cr(VI) detection in water samples in just 3 min. A recent report from Xuan et al. detailed the application of ML in the data analysis of perovskite/metal oxide core/shell nanocrystals-based methanol sensors with potential applications in air quality monitoring and disease screening.^[169]^ RF and Adaboost algorithms were utilized for methanol identification, with the RF-based model being the most effective for confirming the high selectivity of the sensor in mixed analyte environments. The authors reported 94% accuracy for the identification of methanol in mixed environments containing alcohols, benzene derivatives, aldehydes, alkanes, and esters. It is important to note here that, as the implementation of ML in materials science becomes more commonplace, it is also driving major advances in the field of NMs for chiral sensing, which will be discussed in Section 6.6.

### 4.3. Catalysis

The integration of ML in materials science is accelerating NM-based catalyst discovery, design, and optimization. ML facilitates rapid screening of catalyst candidates at a rate that is unattainable by traditional experimentation or computational methods.^[170–172]^ Furthermore, the power of ML is enabling researchers to uncover a deeper understanding of NM catalytic activity and mechanisms.

Several recent studies have detailed how ML can be used to minimize the DFT computational cost of large-scale modeling of NM-based catalysts. Bunting et al. reported the use of an equivariant NN potential, trained using DFT calculations, to understand and predict the behavior of single-atom alloy NP catalysts for propane dehydrogenation.^[173]^ This method allowed for a much larger-scale study on the entire NP to fully understand the catalytic behavior, in comparison to DFT which is limited to much fewer numbers of atoms. A recent report from Bang et al. used a graph convolutional NN to enable exploration of the stability of metallic platinum NPs in electrocatalysis.^[84]^ Pourbaix diagrams—plots of possible thermodynamically stable phases—could be built for NPs measuring several nanometers in diameter, involving thousands of atoms, which is near impossible with DFT alone due to speed and cost.

The use of ML in the field of catalysis is particularly promising for new catalyst discovery. Kim et al. utilized a Pareto active learning model to investigate hydrogen and oxygen evolution reactions of bifunctional alloy catalysts for overall water splitting.^[174]^ The framework, based on supervised Gaussian process regressors, was initially trained using experimental overpotential data, and iteratively enhanced to discover several promising water-splitting catalysts. Recent work from Pillai et al. applied ML to design Ir-free trimetallic electrocatalysts for ammonia oxidation, reducing reliance on precious metals.^[175]^ Graph NNs, trained with data from DFT calculations, rapidly predicted catalyst reactivity, stability, and synthesis ability. A promising Ir-free ternary alloy nanocube was predicted by the graph NN, which was subsequently synthesized and experimentally verified to have excellent activity for ammonia oxidation. Jiao et al. employed a combined DFT/ML approach using a sure dependence screening and sparsifying operator (SISSO) and VASP applications to swiftly screen MXene electrocatalysts for C–N coupling reactions.^[176]^ The use of ML allowed them to rapidly assess 162 MXene candidates, and identify an optimal C–N coupling electrocatalyst (Ta_2_W_2_C_3_), confirmed by DFT calculations.

It is worth mentioning that, while the exploration of NMs for a wide variety of catalytic processes has been explored in depth over numerous decades, an emerging but exciting prospective application of chiral NMs is in the field of asymmetric catalysis. Chiral NMs can be applied in asymmetric catalysis via functionalization of NP catalysts with chiral ligands, by using NMs that exhibit structural chirality, or by the effect of chiral microenvironments.^[177–181]^ While recent reports highlight some of the strides ML has made in the field of asymmetric catalysis,^[182,183]^ to the best of our knowledge there are no reports of ML-assisted discovery of chiral NMs for this application. Looking forward, we envision that the advancement and increasing widespread use of ML will revolutionize the design and utilization of chiral NMs in the field of asymmetric catalysis.

### 4.4. Biomedical Applications

Recent research on NMs in healthcare has led to exciting applications in areas such as wearable electronics, precision treatments, and nanosensors for early disease detection.^[184,185]^ The integration of nanotechnology and ML shows great promise for accelerating discoveries and advancing the use of nanotechnology in medicine.^[186]^

A key application of ML in the biomedical field is enabling the automatic analysis of the read-out of nano-bio-sensors. For instance, Liu et al. developed an intelligent wearable diagnostic tool using a piezoresistive nanocomposite pressure sensor to monitor patients’ pulse signals.^[187]^ An intelligent algorithm was developed to recognize and extract key features of the pulse signal, assisting doctors in making diagnoses. This data was used to train a ML model, achieving a 97.8% success rate in recognizing unique pulse signals without the need for a doctor. Another study by Xu et al. utilized DL for the potential improvement of liver cancer treatment.^[188]^ They developed NanoBeacon.AI, a CNN based on the AlexNet architecture, to be used in conjunction with a nanodiamond-supported biosensor. This framework allowed for rapid prediction of patient sensitivity to a therapeutic within a day, compared to traditional testing which takes three days. AI-assisted sensors are also being developed for automated analysis of complex biological data for diagnostic purposes. Diao et al. used ML-assisted SERS approach for cancer detection using a plasmonic 3D gold NP membrane.^[189]^ Their combined PCA/LDA-based approach was used to distinguish between multiple cancer cell lines with 91.1% accuracy. Furthermore, this approach was used to shine light on the therapeutic mechanism of Doxorubicin using the MCF-7 cell line, demonstrating how ML can enhance our understanding of the efficacy of chemotherapeutic agents.

Another significant advantage of implementing ML in nanomedicine is the ability to rapidly screen drug delivery candidates on a massive scale, which is challenging through physical experimentation or simulation. Reker et al. used a high-throughput combinatorial experimental/ML approach to design NPs formed via the co-assembly of small molecules and drugs.^[107]^ Their RF approach screened 2.1 million potential small-molecule/drug pairings leading to the identification of 100 novel drug NPs with therapeutic actions ranging from asthma to cancer and antiviral drug delivery.

While extensive research has been conducted on chiral NMs for biomedical applications in recent years, the untapped potential of ML in this area remains an area for exploration and innovation.^[190,191]^ It is anticipated the crossover of the fields of ML and chiral NMs could lead to breakthroughs in areas such as precision medicine, biosensing, and targeted drug delivery.

### 4.5. Electronics, Optics, and Human–Machine Interfaces

The integration of ML with NM research is expected to kick-start a new era of possibilities in the field of electronics. By exploiting the powers of ML in pattern and trend recognition and optimization, the rapid acceleration and optimization of NM-based electronic devices and human–machine interfaces are anticipated.

Recent work from Abroshan et al. introduced a novel method to enhance the performance and efficiency of quantum dot-based light-emitting diodes (QLEDs) via ML-enabled high-throughput screening of hole transport materials (HTMs).^[192]^ Initially, the authors generated a large library of potential HTMs from both literature and commercial sources. A synergistic DFT/MD/AL approach facilitated rapid screening of over 8000 HTM candidate molecules, creating a multi-parameter optimization metric to take into account the complexity of the optoelectronic property-performance relationship, reducing computation time by a factor of 18 compared to traditional DFT-based screening approaches.

The application of QDs in solar cells is also an extensively researched area that is likely to be subject to further advances in the age of ML. Ren et al. recently reported a strategy to improve the efficiency of crystalline silicon solar cells, using ML in the analysis of QD doping for spectral down-shifting.^[193]^ The authors used a multivariable linear regression algorithm, programmed in TensorFlow 1.0, to model and fit the absorption spectra of mixed-size QDs to the solar spectrum. Their ML-enabled approach predicted a theoretical increase in c-Si solar cell energy conversion efficiency from 18% to 20.9%, as a result of the inclusion of a QD-doped down-shifting layer.

Significant efforts have been devoted to the use of ML in furthering the applications of triboelectric nanogenerators (TENGs), specifically in decoding the generated electrical outputs and converting them to useful, human-readable information. These TENGs show particular potential for the next generation of human–machine interfaces, that is the conversion of body signals to language, as highlighted more in-depth in a recent review from Ji et al.^[194]^

**Figure 5.**
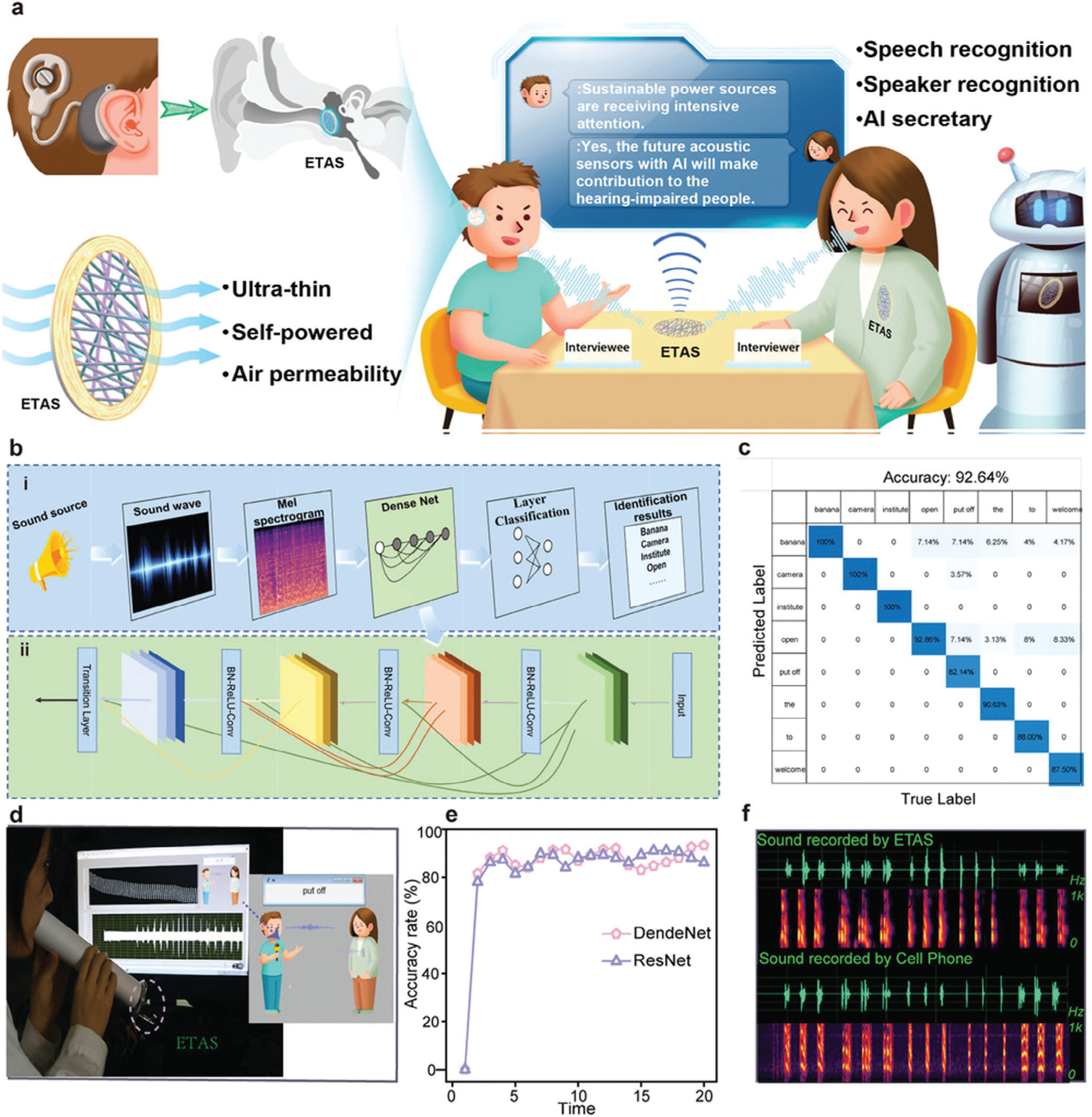
Application of the TENG sensor for voice-text conversion. a) Proposed wearable sensor with hearing aids to allow a hearing-impaired person to interview. b) Schematic diagram of the voice-to-text conversion process using the sensing module. c) The confusion map for ML outcome. d) Real-time voice-to-text conversion demonstrated by the sensing module. e) Accuracy comparison of the DenseNet and ResNet. f) Recorded sound wave information and sound spectrograms recorded by the TENG device and a cell phone. Reproduced with permission.^[195]^

A ML-assisted self-powered TENG acoustic sensor, using silver-coated nanofibers, was developed recently by Jiang et al. with applications for the hearing impaired.^[195]^ A dense convolutional network (DenseNet) was chosen as the optimum algorithm for speech-to-text generation using the acoustic sensor, reaching over 92% voice recognition accuracy after 70 rounds of training. Both the working principle of the sensor, as well as the ML architecture, are represented in the schematic shown in Figure 5.

An et al. developed a DL-enabled intelligent wearable neck sensor, based on carbon-doped silicon-based TENGs, for monitoring posture and maintaining cervical health.^[196]^ A CNN-based model was developed for the task of recognizing the different types of neck movement and did so with over 92% average accuracy. A very recent report from Das et al. details the potential of ML-assisted Au-gC_3_N_4_-ZnO-based TENGs in full-body motion detection using RF, NN, and SVM algorithms, with motion detection accuracy of up to 100%.^[197]^ Wearable bioelectronics have the potential to be revolutionized with the aid of ML, as demonstrated by Kwon et al.^[198]^ Their work details the development of printed nanomembrane hybrid electronics (NHEs) based on functionalized conductive graphene. Data obtained from hand gestures and muscle flexions made by a subject when wearing the NHE were used to train KNN and CNN algorithms to create a human–machine interface, which enabled the user to externally control drones, RC cars, and PowerPoint presentations using various hand movements.

### 4.6. Evaluation and Prediction of Nanotoxicity and Nanosafety

As discussed thus far in this section, it is clear that NMs have found many potential and successful applications, which are anticipated to grow exponentially with the aid of ML techniques, both for automated analysis of complex data, as well as for materials design. As the applications of NMs and their integration into everyday objects continue to grow rapidly, the potential health impacts of this increased exposure to NMs must be carefully considered. If we, as researchers, do not devote sufficient attention and efforts to the analysis of the safety of NMs, this could have huge implications for human and environmental well-being, and the further commercialization and widespread applications of NMs may be significantly limited. As such, extensive research has been undertaken in the field of nanotoxicology in recent years.^[199]^ The substantial volume of data generated from years of research presents exciting opportunities for the use of ML in the prediction of NM toxicity and addressing environmental concerns of NMs.

**Figure 6.**
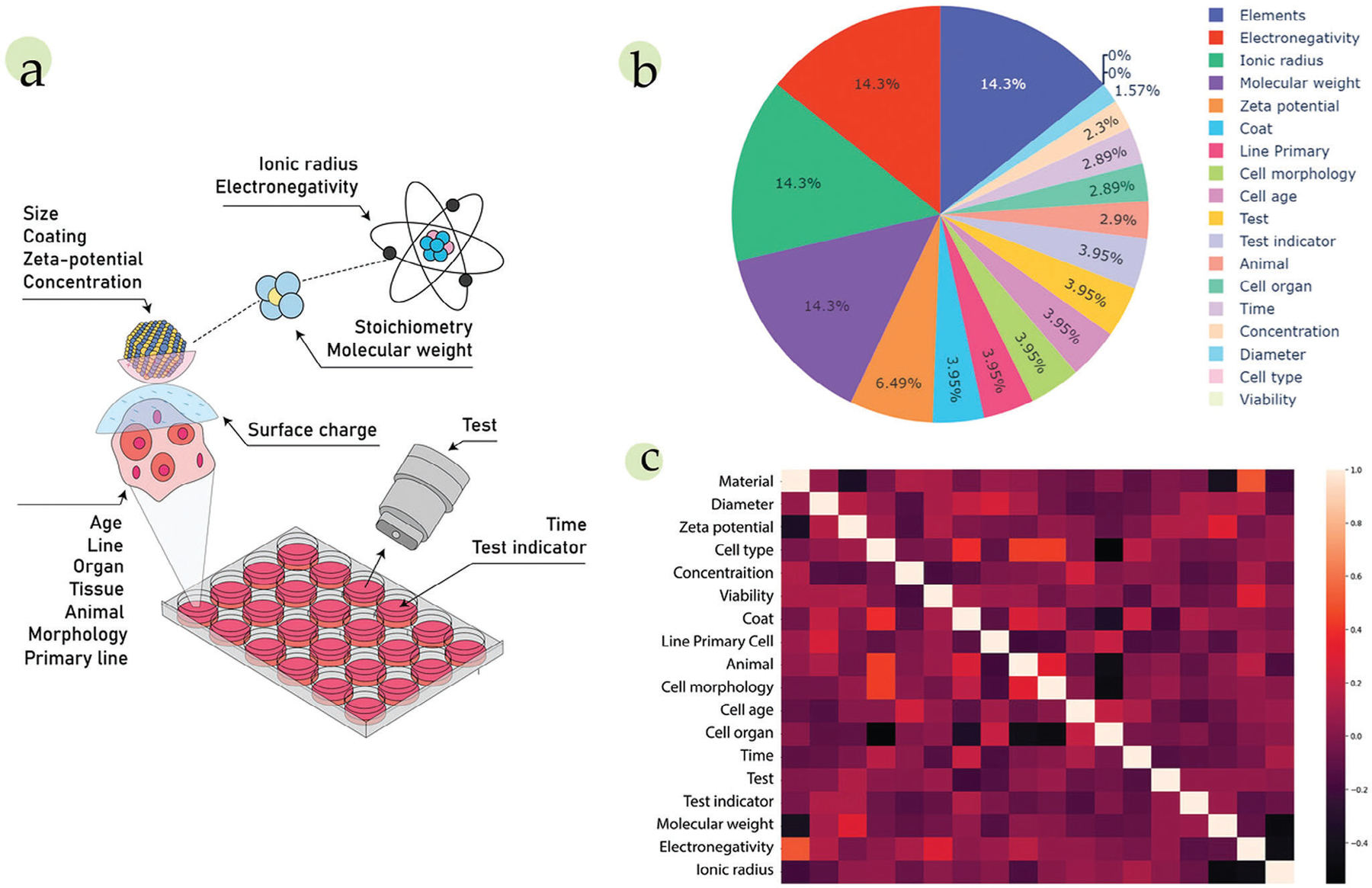
a) Schematic view of model parameters, b) gap statistics over the dataset variables, where the total number of single missing values is 51,666 out of 145,368 values in the initial dataset; c) correlation matrix on the processed dataset (two axes are identical, diagonal cells represent feature self-correlation). Reproduced with permission.^[200]^

Recent work from Shirokii et al. demonstrated a ML-based approach for quantitative prediction of inorganic NMs in vitro cytotoxicity.^[200]^ A database of over 8000 unique samples was created by combining existing datasets with data manually extracted from research articles to train and test 40 ML models for cytotoxicity prediction. For example, the LightGBM model exhibited the highest prediction accuracy and speeds across a range of concentrations, cell lines, and NM types. This study showcases the creation of a valuable tool for researchers to design safe, non-cytotoxic NMs, as summarized in the schematic in Figure 6.

Gakis et al. built one of the largest databases on metal and metal oxide NP toxicity to date, to train a ML model using SVM, KNN and RF algorithms to predict their toxicity toward a variety of cell types, including human, mammalian, fish, and plant cells.^[201]^ Using a testing dataset previously unseen to the ML model, they could predict the toxicity of metal and metal oxide NPs toward a variety of cells, with accuracies of over 90%.

Cadmium-containing quantum dots (Cd-QDs) are of particular safety concern, especially considering their diverse applications such as in QLED-based devices, solar cells, and prospective uses in medicine. Oh et al. mined the literature to compile a database on Cd-QDs cytotoxicity by extracting relevant cell viability data from 307 publications.^[202]^ Using RF regression models to identify trends in the available data, the authors deduced that several factors including QD diameter, surface chemistry (ligands, shells, surface charges, etc.), and exposure time have significant implications for cytotoxicity. More recently, Yu et al.^[203]^ took a similar approach, utilizing LightGBM models to understand Cd QDs cytotoxicity, and coming to very similar conclusions as Oh et al. Moreover, the authors took this study one step further by utilizing SHAP to interpret ML predictions, which is an essential consideration for regulatory and policy-making decisions on NM safety. Martin et al. also utilized SHAP to explain the toxicity of amorphous silica (SiO_2_) NPs as predicted by a categorical boosting (CatBoost) model.^[204]^ The authors mined the literature to create a SiO_2_ NP toxicity database from 115 publications, taking into consideration the implication of NP-corona complexes on toxicity, a facet that is often overlooked in similar studies. Their model predicted key factors in SiO_2_ NP cytotoxicity, acting as a set of guidelines for researchers in the development of safe SiO_2_ NPs by appropriate surface functionalization and the use of low concentrations.

Thus, it is clear that ML shows huge potential in predicting the safety of NMs, thus guiding researchers and policymakers alike. In particular, there remains a significant gap in the field for the development of ML models to predict the toxicity of chiral NMs, even though chirality governs many biological processes and has huge implications for potential toxicity.^[205]^ However, for this goal to be realized, and for the use of ML to become the status quo when considering the safe design of NMs, more strides need to be made in ensuring easy access to large, open-access, ML model-friendly datasets on NM toxicity and safety. As discussed in great detail in a recent review from Yan et al., while there is a large number of available databases on NM toxicity, more efforts need to be focused on ensuring these databases are model-friendly before the full potential of ML can be fully harnessed for nanotoxicology and nano-safety research.^[64]^

## 5. Chiral Nanomaterials

Chirality is one of the most fascinating occurrences in the natural world, and it is one of the most important factors in biomolecular recognition. Chiral compounds play a very significant role in chemistry, biology, pharmacology, and medicine. Chirality has also been envisaged to play an important role in nanotechnology.

**Figure 7.**
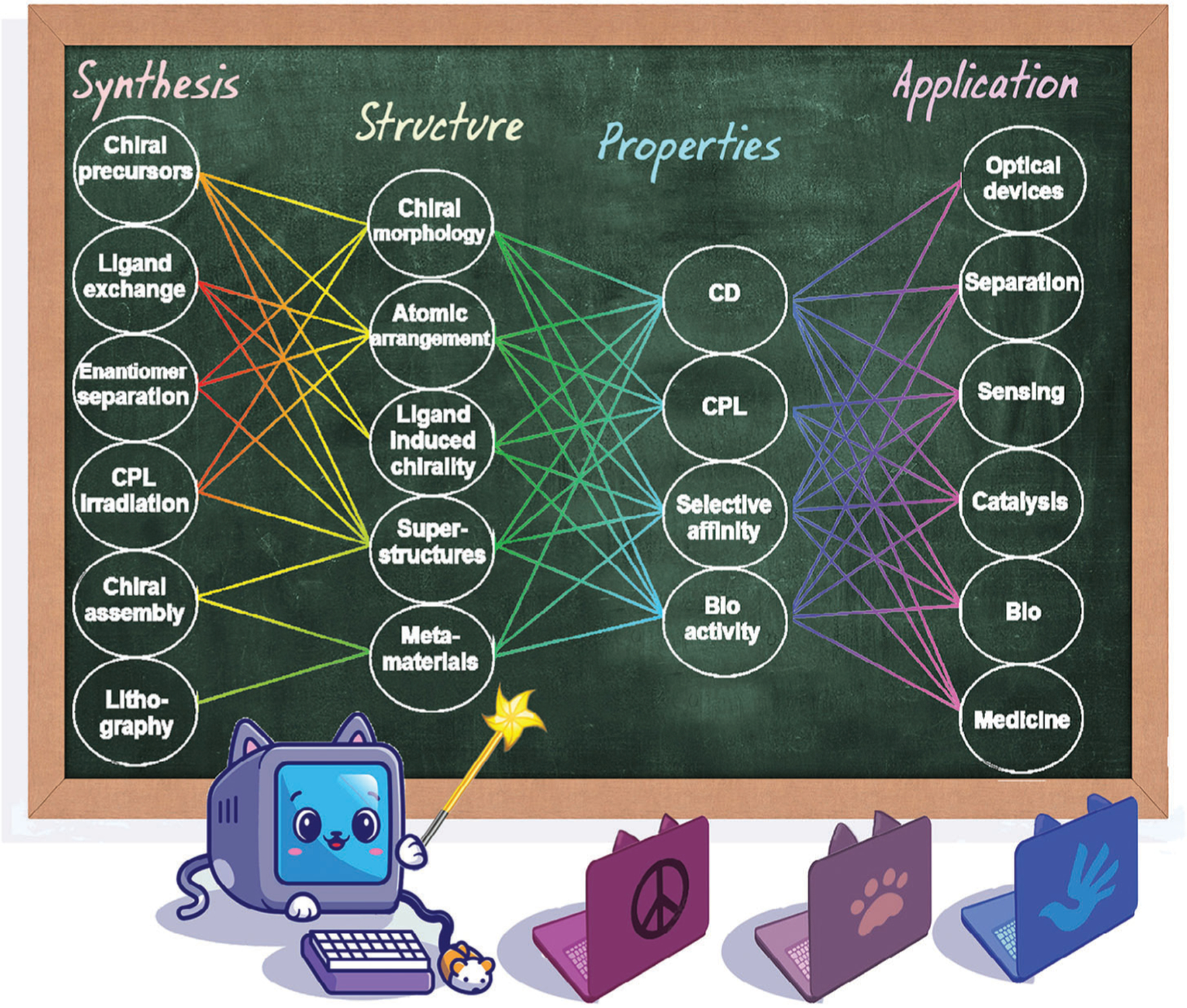
Schematic representation of the connectivity between synthesis, structure, properties, and applications of chiral nanomaterials. (The neural network diagram is used for visualization purposes only and does not correspond to any real deep learning algorithm). This figure has been designed using assets from Freepik.com. Blackboard designed by kjpargeter.

In recent years, there has been a notable shift in attention toward inorganic chiral NMs, due to their unique optical and biomimetic properties, as well as a plethora of proposed and realized applications.^[181,206–208]^ However, this surge in interest in chiral NMs has not been supported or advanced by ML in the same way that general NM development has. To drive further advancements in the field of chiral NMs, researchers need to embrace the power of ML that has already been demonstrated for NMs, in particular the ability of ML to predict structure–property–application relationships, as well as optimize synthetic parameters and even discover new materials. Herein, we will briefly discuss the structural features, synthetic approaches, properties, and applications of chiral NMs, as well as provide researchers with some insight into the use of ML analysis for the investigation of their complex interconnection, as shown in Figure 7.

### 5.1. What Is Chirality?

Chirality is the absence of mirror symmetry in an object. Imagine an object with distinct left and right forms, like our hands. These forms are mismatched in 3D space and are termed enantiomers. The majority of organic molecules in biological organisms are chiral. Enantiomers of these chiral substances, including drugs, interact with biological molecules based on the “lock-key principle,” and as a result exhibit different biological activity, including toxicity.^[23,24,209]^ Notably, over 80% of current drugs and other synthetic biologically active substances are chiral. From this point of view, further development of enantioselective synthesis and catalysis, chiral separation, and sensing are all of extreme importance. Additionally, consideration of chirality is crucial in the design of new non-toxic, and sustainable materials.

Another characteristic property of chiral substances is their interaction with circularly polarized light (CPL). Enantiomers absorb left and right polarized light to varying degrees, i.e., they exhibit chiroptical activity, which is reflected in the circular dichroism (CD) spectra and asymmetry factor (*g*-factor) spectra. Typically, natural chiral compounds exhibit low optical activity and circularly polarized emission, most often occurring in the UV region. By contrast, chiral nanostructures can exhibit huge CD values in the visible region^[210,211]^ and high circularly polarized emission intensities, which can span a wide wavelength range across the visible and NIR spectrum, presenting opportunities for applications in optoelectronics.

Furthermore, chiral NMs can enantioselectively interact with chiral molecules and biomolecules, which leads to changes in their CD spectra—a behavior that can be exploited for chiral sensing. These and other properties make chiral NMs an important area of study. For interested readers, more detailed information on chirality and chiral NMs can be found in the cited reviews,^[24,190,207,212–214]^ including more specific topics, such as plasmonic particles,^[215–219]^ carbon dots,^[220]^ magnetic NMs,^[221]^ perovskites,^[222,223]^ quantum dots,^[26]^ chiral assemblies,^[216,218,224–226]^ CPL emitters,^[214,221,224,227,228]^ biological applications,^[23,191,217,229,230]^ catalysis,^[231,232]^ chiral spectroscopy,^[221]^ and intrinsic chirality.^[28]^

Since the main application of ML in nanotechnology, including the field of chiral NMs, is the analysis of synthesis-structure–property–application relationships, it is necessary to briefly discuss relevant aspects of chiral NMs.

### 5.2. Chirality in Nanomaterials

The origin of chirality in NMs can vary widely and can appear in both single particles and NP assemblies. Frequently, these systems possess several types of chirality at once, all of which can influence each other. For a more comprehensive description and classification of NM chirality, readers may refer to the cited review from Ma et al.^[207]^ Here we aim to describe the main types of chirality that are currently being investigated using ML and give comments on which other types of chirality ML can be used.

#### 5.2.1. Chiral Shape of Individual NPs

##### Chiral Particle Morphology:

The most apparent form of chirality in NP is the chiral shape or 3D geometry of the particle. These NMs can take on various shapes, including coils,^[233]^ helicoids,^[210,234]^ propellers,^[211,235]^ triskelions,^[210]^ gammadions,^[236]^ etc. The degree of shape chirality can be determined by the Hausdorff chirality measure and Osipov–Pickup–Dunmur index,^[211,237]^ which may be used as parametric descriptors for ML.

In recent years, a significant number of works have been published on the synthesis of plasmonic NMs with a wide variety of chiral shapes.^[210,211,215,219,234–236,238–247]^ Some of them have displayed remarkable *g-*factor values,^[210,211]^ up to 0.57.^[210]^ Often, slight adjustments to synthetic conditions may lead to significant changes in NP shape and optical properties.^[210]^ Analysis of the synthesis—shape—*g*-factor relation is an ideal task for ML in order to maximize the observed *g*-value.

For other materials, the chiral form is less typical; nevertheless, chiral nanoceramics and semiconductor particles are known, for example, carbon nanocoils.^[233]^ Folded 2D MoS_2_ sheets were also demonstrated to exhibit optical activity.^[248]^

##### Chiral Atomic Arrangement:

NPs are not perfect, ideal geometric objects. NPs often have asymmetric faces, edges, and vertices, as well as both surface and bulk defects and dislocations present in the crystal structure. Chiral arrangements of chiral defects^[249,250]^ or distortions^[251–254]^ of the crystal lattice (e.g., screw dislocations)^[255]^ can in fact induce chiroptical activity in NPs. The use of chiral ligands, either in situ or by post-synthetic treatment, increases the probability of generating chiral defects and distortions.

Some materials, such as gold and copper, have high Miller-index facets with intrinsic chirality due to the presence of the chiral terraces, or kink sites.^[244,256,257]^ Some materials possess intrinsically chiral crystal lattices, such as 𝛼-HgS, selenium, and tellurium.^[258]^ Nanotubes can also acquire a chiral atomic arrangement, which depends on the direction in which a 2D nanosheet is rolled up to form the nanotube, as is the case for carbon nanotubes (CNTs).^[259–261]^ It is indeed possible to visualize this chiral atomic arrangement with high-resolution TEM (HR-TEM), however manual identification of chiral patterns from images is a challenging task, one which ML can tackle. Furthermore, properties of NPs with chiral atomic arrangement can be predicted by computer simulations. The use of ML significantly decreases the cost of such simulations.

##### Ligand-Induced Chirality and Influence of NP on Chiral Molecules:

Chiral ligands and NPs have a sophisticated mutual influence on each other’s properties. While chiral ligands can induce optical activities in achiral NPs, binding to the NP surface can also change the chiral properties of the ligand itself.^[27]^

Due to the high polarizability of the NMs, an asymmetric redistribution of electron density can occur under the influence of the field of chiral molecules.^[207,262]^ This influences the electronic structure of NPs, leading to a change in the UV–vis and CD spectra.

The HOMO electron levels of molecules can interact with plasmons in metal NPs^[263,264]^ and excitons in semiconductor QDs,^[26,265–267]^ and carbon dots^[268]^ leading to coupling and splitting of energy levels and the appearance of a CD signal in the region of these transitions. If the molecule has multiple anchor groups, the binding mode to the NP surface determines the spatial configurations of the molecules, which affects both the chiral properties of the NPs and the molecules themselves, as well as their electronic interaction. Depending on the binding mode, even the same enantiomer can induce an opposite CD signal in a NP.^[26,265,269]^

#### 5.2.2. Chiral Assemblies of Nanoparticles and Metamaterials

Even intrinsically achiral NPs can exhibit chiral properties if arranged in a chiral manner, which are commonly referred to as chiral assemblies. Chiral assemblies,^[207,216,218,224–226,270]^ which may consist of chiral or achiral NPs, are capable of demonstrating significant optical activity, arising from the strong electromagnetic coupling between the building blocks. The most reported types of chiral arrangement of NPs into chiral assemblies include:

- Pyramids consisting of NPs of different sizes or compositions located at the vertices and most often connected by deoxyribonucleic acid (DNA). This kind of chirality bears a resemblance to molecules with a tetrahedral carbon chiral center.^[207]^
- Twisted pairs of nanorods, nanosheets, or other anisotropic NPs. The chirality originates from the angle of rotation between particles.^[271]^
- Helices (and chains) can consist of both spirally arranged particles and stacks of rods or platelets rotated at a certain angle.^[272,273]^
- Chiral nematic structures similar to liquid crystals.^[224]^
- Hierarchical chirality, in which the chirality of elements is translated into the chiral geometry of the assembly, and then in turn to the chiral structure of a higher level. This is similar to how amino acids in proteins form alpha helices, and then tertiary and quaternary structures.^[163,213,274,275]^

Special attention should be given to metamaterials (MMs),^[276–278]^ which consist of chiral or asymmetrically arranged particles periodically repeating in space with a period shorter than a wavelength of light. When interacting with electromagnetic radiation, they act as an assembly, giving a synergistic effect greater than the sum of the effects of individual elements. Such materials can possess giant CD values.

Optimization of assembly structure and the nature of building blocks is an important task for ML. ML can be used for finding relations between the structure and properties of real and simulated ensembles, and automated prediction of structures with desirable performance.

### 5.3. Synthetic Strategies for the Preparation of Chiral Nanomaterials

The analysis of synthetic conditions, structural parameters, and properties are among the main subjects of research for ML in nanotechnology. Therefore, here we will consider some of the main strategies for producing chiral NPs, and comment on how the application of ML may be suitable for progressing this area.

#### 5.3.1. Synthesis in the Presence of Chiral Molecules

Chiral molecules can affect NPs in a number of very complex ways. In almost all cases, when NP synthesis is carried out in the presence of chiral ligands, this results in the induction of some type of chirality. Depending on various factors, this can lead to the formation of a chiral shape (mostly for plasmonic particles),^[210,211,215,219,234–236,238–247]^ chiral defects in the volume and on the surface of the NP,^[208,250]^ embedding or intercalation of chiral molecules between layers of layered materials (e.g., perovskites and layered double hydroxides)^[279]^ and in the case of chemical bonding with the NP surface, hybridization of electronic energy levels^[26,263–268]^ and distortion of surface atoms.^[251]^

The concentration of chiral agents, as well as other chemical precursors, is of great importance for the induction of chirality. By slightly varying the concentrations of the precursors, one can produce chiral particles of vastly different shapes and morphologies. For example, over the past number of years, the production of chiral gold particles with a wide variety of shapes, based on similar synthetic methods with only slight variations, has been intensively studied.^[210,211,215,219,234–236,238–247]^ This is a seed-mediated synthesis in which gold salts are reduced with ascorbic acid in the presence of surfactant stabilizers with the addition of a very small (usually nanomolar) amount of a chiral additive, often cysteine or cysteine-containing peptides, and also halides^[144]^ or oligonucleotides.^[241]^ Gold has high Miller-index facets which are intrinsically chiral.^[241,256,257]^ Such surfaces can exhibit different affinities for enantiomers of chiral molecules, and this is often used as a driving force to promote selective asymmetric crystal formation due to the preferential growth of one of the enantiomeric facets.^[241,244,256,257]^ NP shape can affect the position and intensity of the CD signal peaks in a complex way. ML can be a useful instrument for studying this complex correlation between the concentrations of precursors, shape, and CD spectra of chiral plasmonic single NPs. It is worth highlighting a very recent study from Choi et al,^[234]^ which reports a ML-based methodology for visualizing the distribution of structural strain and the high-Miller-index planes constituting the concave chiral gap of gold 432 helicoid by Bragg coherent X-ray diffraction imaging, which can be a very useful technique for investigation of the chiral shape of NPs.

#### 5.3.2. Post-Synthetic Functionalization with Chiral Molecules

Chiral substances adsorbed on the surface of even achiral NPs can induce optical activity. In many instances, NP synthesis cannot be carried out in the presence of chiral agents, for several reasons. For example, QDs are often synthesized by the hot-injection method, which is conducted at high temperatures in a hydrophobic environment. Another potential reason for avoiding the introduction of chiral molecules during NP synthesis is that chiral substances can negatively impact the synthesis, leading to the formation of mixtures containing undesirable forms of NPs, as well as unwanted side products. In these cases, NPs can instead be functionalized with chiral substances after synthesis. Post-synthetic ligand exchange is widely used to induce chirality in semiconductor QDs.^[26,265,280]^ Many factors impact the shape and magnitude of the CD spectra, including the size, shape, and structure of the particle, the type and binding mode of the molecule, pH, ligand concentration, etc.^[265]^ These factors can lead to a significant shift of the peaks, up to a complete “flip” of the signal to the opposite sign. This is discussed in detail elsewhere in an in-depth review from some co-authors of this work.^[26]^ Automated analysis of ligand-induced optical activity for various chiral QDs using an intelligent machine was recently reported by Liu et al.^[281]^

#### 5.3.3. Separation of Enantiomeric Nanoparticles

In some cases, during synthesis, both enantiomers of NPs (racemic mixture) can be formed. This is typical for CNTs,^[282]^ chiral twisted nanowires, helicoids and coils, and crystals possessing an intrinsically chiral lattice, such as HgS.^[207]^ Also in any conventional synthesis, particles with chiral defects can be incidentally formed.^[208]^ Such enantiomers can be extracted from the mixture with the use of chiral substances, for example, proteins or cysteine.^[283]^ To separate CNTs, density gradient centrifugation, chromatography, and two-phase separation methods are commonly used.^[282]^

#### 5.3.4. CPL Irradiation

Irradiation with circularly polarized light during NM synthesis can lead to the formation of chiral crystals,^[237]^ or can be used to selectively enhance chiral properties initiated by ligands,^[210,211,284]^ due to a chiral redistribution of the electric field in the growing crystal. In some cases, CPL irradiation during synthesis can increase the *g-*factor by up to 40%.^[214]^

#### 5.3.5. Nanolithography

Nanolithography is a powerful technique, which enables the production of particles possessing very complex morphologies, with relative ease and high precision.^[285,286]^ Nanolithography is one of the main methods of producing metamaterials,^[285,287]^ to which most of the ML-based studies on chiral materials are devoted. These studies mainly use computer-simulated data, without resorting to the fabrication of real materials, but optimized structures can be easily discovered and subsequently fabricated by nanolithography. Metamaterials will be discussed in more detail in the corresponding section.

#### 5.3.6. Chiral Assemblies and Superstructures

Chiral NP superstructures can be obtained using several methods: templates,^[216,218,224]^ external forces,^[225]^ and self-assembly.^[163,207,216,225,226,270]^ The most straightforward method to fabricate chiral nano-assembles is using organic templates,^[216,272,273]^ with DNA origami being the most widely studied.^[288]^ Through complementarity, DNA binding allows short strands to act as staples, connecting long strands. Thus, DNA can self-assemble into various templates, which can be pre-designed, forming a nanoscale scaffold for NPs of any arbitrary shape,^[225]^ including pyramids, helices, or linkages between two rods. Fabrication can be scaled up due to PCR replication of DNA. Synthetic peptide conjugates, cellulose NCs, and liquid crystals^[218]^ are other versatile platforms for constructing inorganic chiral superstructures. Chiral external fields and forces, such as CPL, magnetic fields, and mechanical forces like vortices, may also be used to induce the chiral arrangement of NPs. Chiral NPs can self-assemble into higher order chiral hierarchical superstructures as a result of particle or ligand asymmetry creating a bias in the particle arrangement.

### 5.4. Properties and Applications of Chiral Nanomaterials

#### 5.4.1. Optics and Electronics

The optical activity is one of the unique properties of chiral NPs, which is intensively studied and exploited for many applications, not only in optical devices, but also for sensing, catalysis, and biomedicine. The vast majority of publications on ML in the field of chiral NMs are related to studies of the CD spectra. Over the past few years, significant progress has been made in the development of NMs with very high *g*-factor values, with single plasmonic particles reaching values of 0.57.^[210]^ It is likely that combining single particles with a large *g*-factor into assemblies or metamaterials may lead to the development of structures in which the *g*-factor approaches the maximum possible value of 2. ML can be useful for designing the parameters of such an assembly.

The ability of chiral NMs to absorb light of one handedness while transmitting light of the opposite handedness, with a tuneable wavelength, can be used to create polarizers, CPL detectors,^[289]^ displays,^[236]^ and microwave-absorbing materials.^[233]^ The interaction of chiral particles with light (even unpolarized) can generate a physical force depending on the particle handedness, which can be used to separate chiral NPs, by moving them in opposite directions or trapping them inside chiral light beams, or may even be used to manipulate chiral nanomachines.

Circularly polarized luminescence is the difference in the emission of the left and right CPL of chiral luminescent materials^[214,226,227]^ and is most commonly reported in materials such as perovskites,^[290,291]^ QDs, carbon dots,^[220]^ and the chiral assemblies of these materials.^[214,224,227,228,290,291]^ Thus, chiral luminescent materials can be used as the foundation of CPL emitters. The use of a magnetic field^[292]^ and plasmonic particles^[293]^ can increase CPL emission intensity.

Non-linear chiroptical effects, such as second-harmonic generation circular dichroism, and optical rotation can be much higher than their linear optical counterparts. For example, plasmonic 432 helicoid III NPs have demonstrated non-linear *g*-factors of up to −1.63.^[294]^ Third harmonic Mie scattering on CdTe helices dispersed in liquid allows for the characterization of the chiroptical properties of NMs with very low sample volumes (1 μL).^[295]^ ML was applied to study T-like shaped chiral metamaterials that exhibit the strongest CD response in the third-order diffracted beams.^[296]^

#### 5.4.2. Separation and Filtration

Chiral NPs bind with chiral molecules according to the “lock-key” principle and have different affinities for different organic enantiomers.^[191]^ This can be due to both the chiral surface of NPs (gold NPs have proven themselves well in this area) as well as chiral ligands on the surface of an otherwise achiral NP (for example, cyclodextrin on magnetic particles).^[297]^

#### 5.4.3. Sensing

The recognition of enantiomers^[191,213,219,298,299]^ by NPs can be achieved due to the enantioselective adsorption of molecules on chiral NPs, which leads to an optical response being generated, for example, a change in the CD signal, luminescence intensity (QDs and carbon dots^[220,268]^), or SERS response^[219]^ (plasmonic particles). The optical activity of a chiral particle can also change upon the adsorption of achiral substances, such as hydrogen, lead ions, or reactive oxygen species (ROS).^[240]^ In addition, a chiral molecule can induce a CD response even in achiral NPs^[268]^ and films. The bulk of studies on chiral sensing are devoted to the adsorption of chiral molecules on gold NPs and metasurfaces, which leads to the appearance of a very strong CD signal in the visible and NIR spectral region and is typically much more intense and red-shifted than that of the molecule.^[219]^ This makes the chirality of the molecule “visible” in CD spectra. This response can be exploited to detect proteins and to analyze their structure. Moreover, plasmonic NPs can enhance not only the Raman signal of a chiral molecule (SERS) but also the Raman optical activity.^[219,221]^ Ultrasensitive DNA analysis can be carried out using NPs functionalized with DNA complementary to the analyte due to the formation of chiral assemblies or changes in existing NP DNA origami superstructures.^[225]^

#### 5.4.4. Catalysis

Enantioselective excess in asymmetric catalysis with the use of chiral NPs^[191,213,219,231,299,300]^ can be achieved due to 1) enantioselective adsorption of precursors; 2) specific conformation and mutual arrangement of precursor molecules on the NP surface, favoring the formation of one of the enantiomers; and 3) selective absorption and translation of external forces, such as CPL.^[214,273]^ Due to the optical activity of chiral NPs, the use of CPL is much more efficient and selective than natural or linearly polarized light due to more efficient absorption and hence the generation of hot electrons.^[214,273]^ In this regard, a large body of work is devoted to asymmetric electrocatalysis.^[190,213,219,231]^ Along with the conventional coating of electrodes with chiral ligands, chiral cavities can be created on the electrodes by etching a metal plate with chiral molecules. Chiral molecules can then be removed, leaving the cavities chiral. Such cavities have selective adsorption to precursor enantiomers and promote a higher oxidation current density. Classic asymmetric organic catalysts such as BINAP can be attached to the surface of particles (predominantly magnetic particles).^[299]^ In this case, the particles act as a support and can be removed from the reaction mixture with a magnetic field. Furthermore, self-assembled NPs can catalyze reactions more efficiently than individual particles due to the synergistic effect.^[301]^

#### 5.4.5. Biological Applications and Toxicity

Chiral NPs can interact enantioselectively with various chiral bioactive substances,^[27,191]^ such as proteins^[297]^ and nucleotides, and affect their properties, for example, enzyme activity, aggregation of amyloid proteins,^[302,303]^ transcription of DNA and ribonucleic acid (RNA). Due to the optical activity of chiral NPs, CPL^[214,303–305]^ can be used to selectively activate NPs with certain handedness, such as inducing reactive oxygen species (ROS) generation for phototherapy^[304]^ or heating for thermotherapy.^[302]^ When combined with an enantioselective interaction with cellular components, CPL can lead to a very strong effect. The effect of ROS generated under CPL irradiation^[214]^ can be used for DNA and protein cleavage, both intracellularly and in vitro.

Enantiomers of NPs have different biological activities,^[191]^ including toxicity,^[23,306]^ antiviral^[305]^ and antimicrobial effect,^[220,306]^ cell uptake,^[235,306]^ gene expression,^[303]^ ROS generation, immune response,^[211,284]^ and growth factor expression. Moreover, NP handedness does not unambiguously determine the effect. Due to enantioselective interaction with many cell biomolecules, such as protein receptors^[211,284]^ and enzymes, as well as RNA, DNA, and polysaccharides, the mechanism of influence on the cell can be very complex and involve multiple stages. For example, in publication^[211]^ the mechanism of enantioselective and highly specific interaction of chiral gold particles with immune cells was studied in detail. Chirality also has a significant impact on nanotoxicity.^[23]^ There are several databases on the toxicity of achiral NMs, which facilitates ML analysis. While the toxicity of chiral NMs has been actively studied in-depth, a unified database does not yet exist, hindering the progress of ML for chiral NMs.

## 6. Machine learning for Chiral Nanomaterials

As has been demonstrated thus far in this review, ML shows huge promise for revolutionizing the field of nanotechnology. Looking forward, we anticipate that the recent strides made in NM research using ML will be made more applicable for chiral NMs in the coming years. In this section, we will discuss some recent advances in the use of ML of chiral NM, as well as provide some outlook for this emerging area of research. The examples of ML methods described throughout this section are summarized in Tables S9,S10 (Supporting Information).

### 6.1. How to Image Chirality?

#### 6.1.1. Electron Microscopy

**Figure 8.**
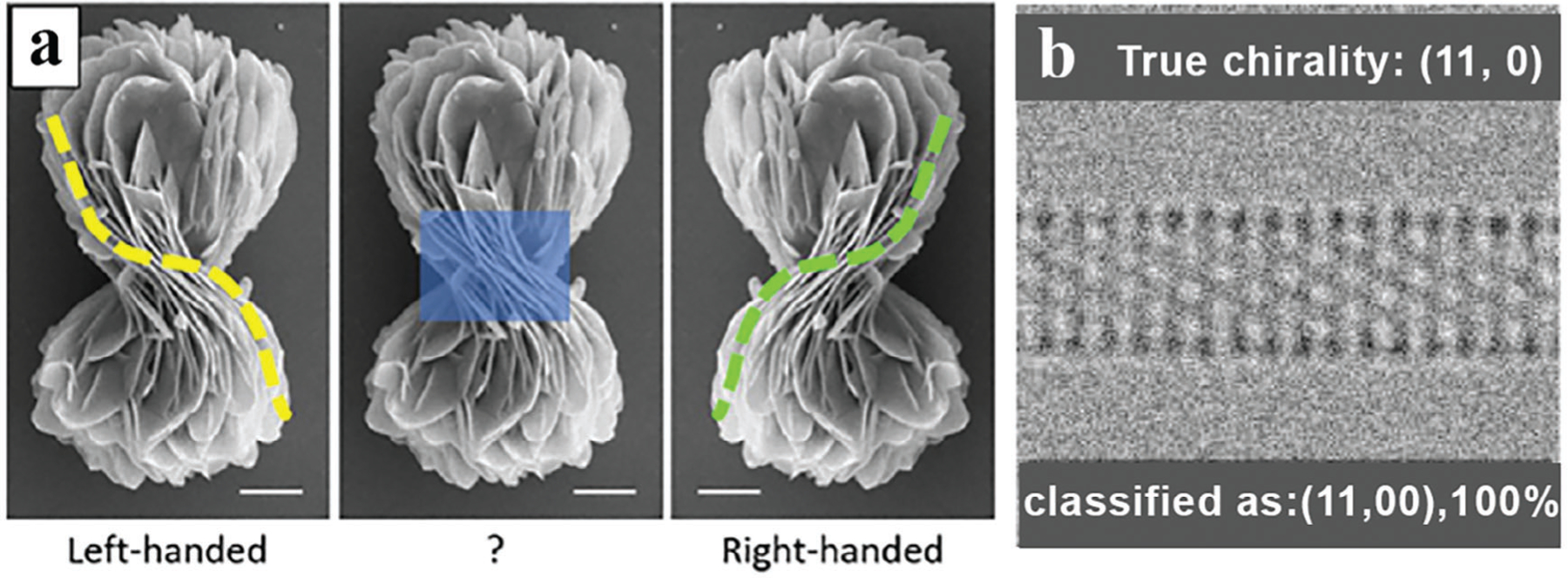
a) Example of classification of left-handed and right-handed chiral bowties. Numbers in the bounding boxes represent the confidence of the model in the proposed class. Reproduced with permission.^[29]^ Copyright 2023, American Chemical Society. b) Examples of assignments of chiral indices of CNTs in the case of simulated HRTEM images. Reproduced with permission.^[261]^

The most direct method to observe chirality in the nanoworld is to capture electron microscopy images of chiral NPs, and subsequently attribute its shape as either left- or right-handed. We refer readers who are interested in the application of ML for electron microscopy analysis to the review.^[46]^ A great example of analysis of NP handedness using such an approach is described by Visheratina et al., where a DL model was used to identify the chiral morphology of twisted bowtie-shaped microparticles based on SEM images, as shown in Figure 8a.^[29]^ Another example of ML-enabled determination of chiral handedness from SEM images was reported by Groschner et al., showing a CNN trained on SEM images was used to automatically determine the handedness of chiral Tellurium NPs.^[307]^

ML is also increasingly being used in TEM analysis of chiral NPs. For example, a CNN model was applied to determine the chirality of CNTs from HR-TEM images by analyzing the atomic arrangement, as shown in Figure 8b.^[261]^ In this regard, close attention should be paid to AtomAI, an open-source software package, which operates on DL principles, for converting HR-TEM images into class-based local descriptors for further analysis.^[132]^

#### 6.1.2. Optical Microscopy

Optical microscopy, especially fluorescent microscopy, is often used for the investigation of optical properties and biological behavior of chiral NMs.^[308,309]^ For example, confocal laser microscopy enables tracking of enantioselective cellular uptake of cysteine-capped CdSe/ZnS QDs^[310]^ and gold NPs.^[235,311]^ ML can significantly simplify the analysis of fluorescent images, especially when dealing with biological samples, as demonstrated by Zhu et al., in which image segmentation using a U-net CNN model helped to resolve long-standing questions on NP transport through tumor vessels.^[312]^

Optical microscopy can also facilitate direct observation of chirality in nanostructures.^[313]^ There is also a report on the handedness determination of single chiral lanthanide-based luminescent NCs using only a single circular polarization component of the emission spectrum.^[314]^ A ML algorithm enabled authors to determine and spatially map the handedness of individual NCs with high accuracy and speed.

#### 6.1.3. Scanning Probe Microscopy

Scanning probe microscopy (SPM) provides tremendous control of condensed matter: single atoms and molecules can not only be imaged with a resolution down to the single chemical bond/electron orbital limit but they can be maneuvered and manipulated with the tip of the microscope.^[315]^ Therefore, SPM might prove to be useful not only for the investigation of properties of chiral NMs but also for the construction of new chiral species on an atomic level. Current research efforts in the field are focused on the construction of fully automated SPM processes.^[316–318]^ Existing AI frameworks possess an algorithmic search of good sample regions and monitor the state of the probe.^[319]^

### 6.2. Simulation of Chiral Nanomaterials

Some of the pioneering works on chiral CdTe NCs^[320]^ and gold NPs^[321,322]^ used DFT simulation to support the hypothesis of the origin of chirality. DFT is routinely used for the simulation of ligand interactions with NP surfaces,^[265,323,324]^ as well as for investigation of the influence of chiral defects on optical activity in crystals.^[249]^ Finding the most stable defective structures is a challenging process, which may be solved more rapidly and efficiently by implementing ML.^[325]^

Recently, DFT simulations were used to investigate the role of cysteine in the evolution of asymmetry of twisted gold nanorods during seeded growth.^[244]^ CPL-mediated generation of chiral gold NPs and their growth patterns were investigated using finite-difference time-domain (FDTD), and semi-empirical DFT simulations.^[211]^ Additionally, FDTD simulation aided in understanding the relationship between 3D morphologies and chiral plasmonic properties of chiral Au nanooctopods.^[235]^

DFT is the most popular method of quantum mechanical calculations, however, other quantum approaches are also often used. An example of alternative quantum chemical approaches for chiral materials is reported by Luo et al.,^[326]^ in which the ground state of the chiral Ag_70_ metal clusters was optimized by the semi-empirical tight binding method (GFN-xTB),^[327]^ in conjunction with the GBSA model for methanol; all excitations were calculated with the simplified Tamm–Dancoff approach.^[328]^

Thus, it is clear that the simulations hold huge power in the study of chirality in NMs. As it was discussed in Section 3.7, the incorporation of ML into the simulation pipeline can reduce computational costs significantly. NNs were trained on data from MD simulations to predict the mechanical properties of chiral single-walled carbon nanotubes (SWCNTs) up to 4 nm in diameter,^[329]^ graphene-reinforced aluminum nanocomposites,^[330]^ and 1H-MoSe2 and 1T-MoSe2.^[331]^ It has been demonstrated that DL provides accurate predictions, comparable with DFT, with the added advantage that thermal fluctuations predicted by DL are smoothed out.^[329]^

### 6.3. Metamaterials

To date, the study of chiral metamaterials (MMs) constitutes the majority of the work reported on ML for chiral materials. MMs are engineered assemblies of structural elements repeating periodically either in 2D (metasurfaces) or 3D space (the so-called meta-atoms) at scales smaller than a wavelength.^[278,332]^ Light irradiation leads to a nonlinear complex distribution of the electric field in the MMs, which in turn results in the emergence of optical properties that are highly dependent on the structure of meta-atoms and their mutual arrangements and are fundamentally different from bulk material and from MM structural elements properties, for example, negative refractive index and negative reflection. MMs are mainly produced by nanolithography, which allows to reproduce materials with any desired shape facilitating the transfer of ML design to practical applications.

Chiral MMs,^[278,332]^ consisting of asymmetric or non-symmetrically arranged elements, have unprecedented chiroptical properties, such as very strong optical activity in spectral range, and nondispersive zero ellipticity. The optical response of MMs can be simulated by calculating the Maxwell function,^[332]^ usually using simulation methods such as the rigorous coupled-wave approach,^[333]^ the finite element method^[334]^ and finite-difference time-domain^[335,336]^ using the COMSOL Multiphysics software package. However, these calculations are time-consuming and resource intensive. ML models trained on simulated data can predict the optical response much faster (on the time scale of seconds instead of hours or days).^[337]^ ML can be implemented in the forward prediction of MM optical properties and for the inverse design of the MMs with desired properties.^[332]^ Published works on the use of ML for MMs mostly describe gold materials, in which the meta-atoms are asymmetric elements of various shapes, mainly built on rectangles. Typically, these are gold particles in a dielectric matrix, but L-shaped holes in a gold film were also studied.^[338]^ The CD signal is usually simulated by the methods described above, although sometimes alternative previously obtained data on MMs behavior is also occasionally used.^[296,333]^ To generate a large amount of data (tens of thousands of data points) for ML training, the optical responses of systems are simulated by varying some parameters with a certain step, for example, size, mutual distance of elements, and rotation angle. In some studies, ML models are trained to modulate the electric field distribution in MMs under light irradiation, including CPL. Additionally, some reported approaches validate their ML models on real objects.^[335,339]^

The most commonly used ML method for this task is DL, specifically and most often CNN and fully connected NN. Pioneering work on the use of ML for the analysis of MM properties from Ma et al. presents a ML model consisting of primary and auxiliary bidirectional networks, assembled by a stacking strategy for increasing both accuracy and functionality.^[340]^ Bidirectional mapping allows one to simultaneously perform forward prediction and inverse design. The primary network analyzes the relationship between structural parameters and reflection spectra of MMs under different polarization irradiation conditions, while the auxiliary network predicts the CD spectra. The two networks are interconnected, allowing them to exchange experiences and knowledge. The work interestingly solved the problem of mismatch of the dimension of structural characteristics of 1 × 5 and spectra of 3 × 201, which leads to inaccuracies in the analysis, especially in the spectra maxima. To address this mismatch, an upsampling module which gradually increases the data dimension was used for the direct prediction, and a CNN for the inverse path. Work from Li et al. reports a self-consistent framework that combines Bayesian optimization and deep CNN (BoNet) algorithms to calculate and optimize electric-field distribution, reflection, and CD spectra of metallic nanostructures.^[335]^ The model was validated on real structures fabricated by electron beam lithography, achieving 82% accuracy of theoretically predicted CD at the target wavelength. Other works also detail the use of fully connected NNs used to predict third-order diffracted CD of a gold array of T-like shaped structures.^[296]^ Work from Ashalley et al. details the use of an end-to-end functional bidirectional DL model with multitask joint learning features to recognize the relationship between yin-yang-shaped gold MM structural parameters and their chiroptical response.^[334]^ To solve the problem of inaccuracy in predicting CD resonances in the local optima typical for all for most DL models, an auxiliary network was used. ML-optimized structure was verified on chiral sensing application. A model-agnostic data enhancement including NN, RF, and SV regression was used by Du et al. to analyze high-order diffracted CD spectra.^[333]^ The algorithm was trained on alternative already studied MM data enabling them allowed to significantly decrease the size of the target problem training dataset. A fundamentally new approach based on RL was used in another report from Chen et al.^[341]^ In their work, RL was used to determine the structures that would be most effective for constructing a ML training dataset, focusing on MMs with strong CD, rather than a search of all possible MM structures, significantly decreasing the computational cost. The details of the studies described above are described in Table S10 (Supporting Information).

The potential applications of chiral metasurfaces are diverse, ranging from enantioselective sensing and imaging to polarization rotators and circular polarizers, to polarization-sensitive meta-holographic displays. The possibilities of using simulated MMs for chiral sensing have also been explored by researchers.^[334,339]^

### 6.4. Carbon Nanotubes

CNTs are among the most widely studied materials which possess intrinsic chirality. CNTs are often classified based on their chirality, which can be defined using the roll-up vector (*n*,*m*), where n and m are integers, or by the chiral angle 𝜃 paired with diameter *d*. These parameters represent the direction along which a 2D sheet of graphene is rolled into a cylindrical shape, resulting in the classification of SWCNTs as armchair (where *n* = *m*, or 𝜃 = 30°), zigzag (*n*, 0, or 𝜃 = 0°) or chiral ((*n*,*m*), *m* ≠ 0, or 0 ≤ 𝜃 ≤ 30°).^[342]^ Furthermore, the SWCNTs can be described as having right-handed chirality if *n* – *m* > 0, and left-handed if *n* – *m* < 0.^[343]^ Automated elucidation and exploitation of the chirality of CNTs via ML are of the utmost importance for developing their applications, as there is a strong structure–property correlation observed in these materials with regard to their electronic, mechanical, thermal, and optical properties.^[260,344]^

**Figure 9.**
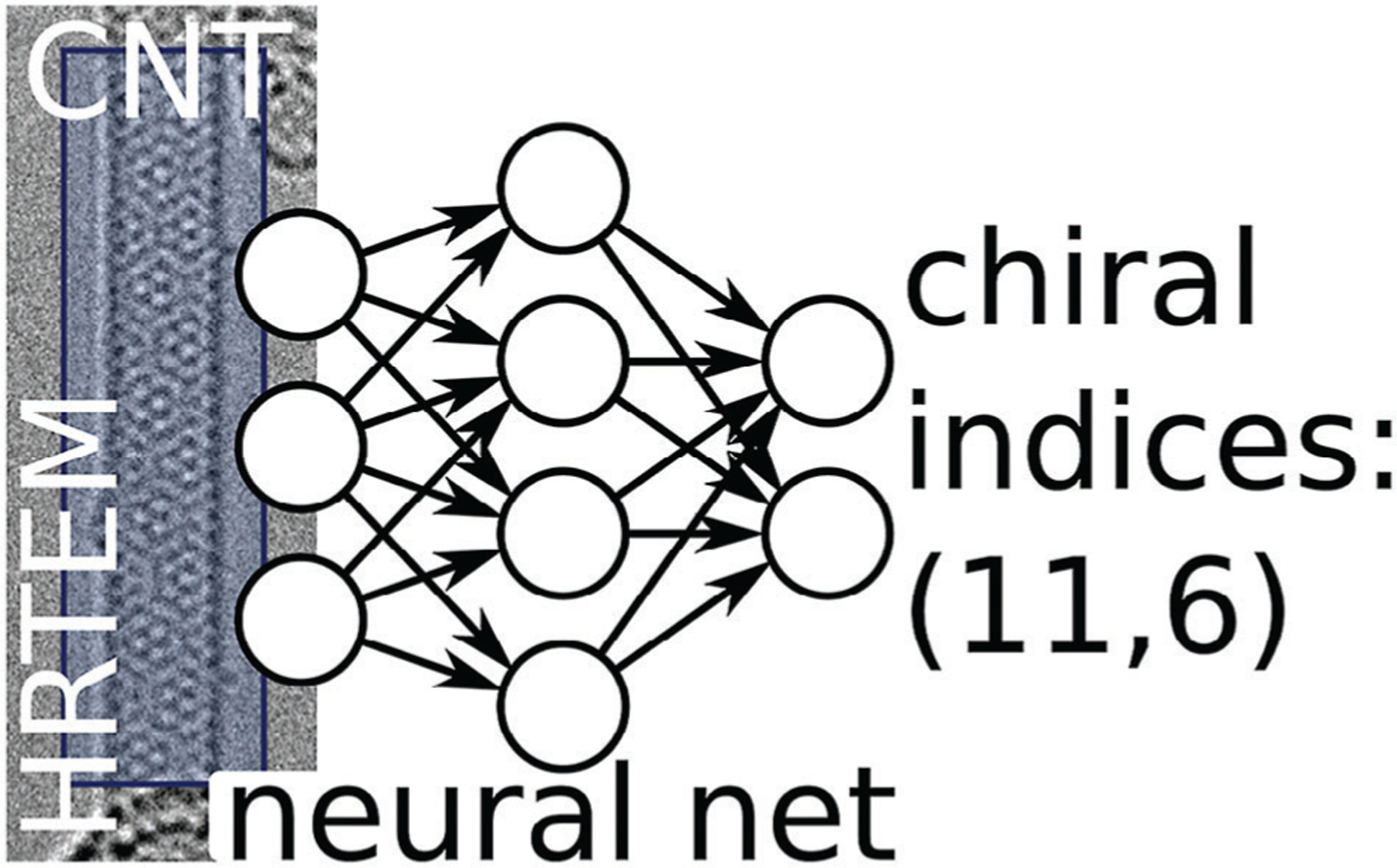
Schematic representing the use of NNs for the determination of chirality in CNTs. Reproduced with permission.^[261]^

Recent reports detail the use of ML for the automation of time-consuming tedious approaches for chirality determination and separation, as well as the prediction of chirality-dependent properties and behavior of SWCNTs. A CNN-based approach was applied for the automated determination of (*n*,*m*) from HRTEM images of SWCNTs, as shown by the schematic in Figure 9.^[261]^ Their approach could predict the chiral indices with 71% accuracy and could be further increased by focusing on SWC-NTs with lower defect densities. An in-depth study of the mechanical properties of SWCNTs was performed with the aid of a sequential dense layered DNN developed by Keras, successfully predicting the strong dependence of the mechanical properties on 𝜃 with high accuracy and smoother results compared to MD simulations.^[329]^ A recent study by Lin et al. employed a trialgorithm-based ML model consisting of RF, NN, and SVM algorithms, in conjunction with experimental studies, to examine the efficacy of using single-stranded DNA to sort SWCNTs according to their chirality, improving the success rate from 10% for the empirical approach to over 90% for the hybrid experimental-ML approach.^[345]^ Finally, not only can ML be used to accelerate the wide-scale applications of SWCNTs, but in return SWCNTs can be applied to advance the field of ML by being used to fabricate neuromorphic devices for improved performance of AI systems.^[346–348]^

### 6.5. “The Laboratory of the Future”

**Figure 10.**
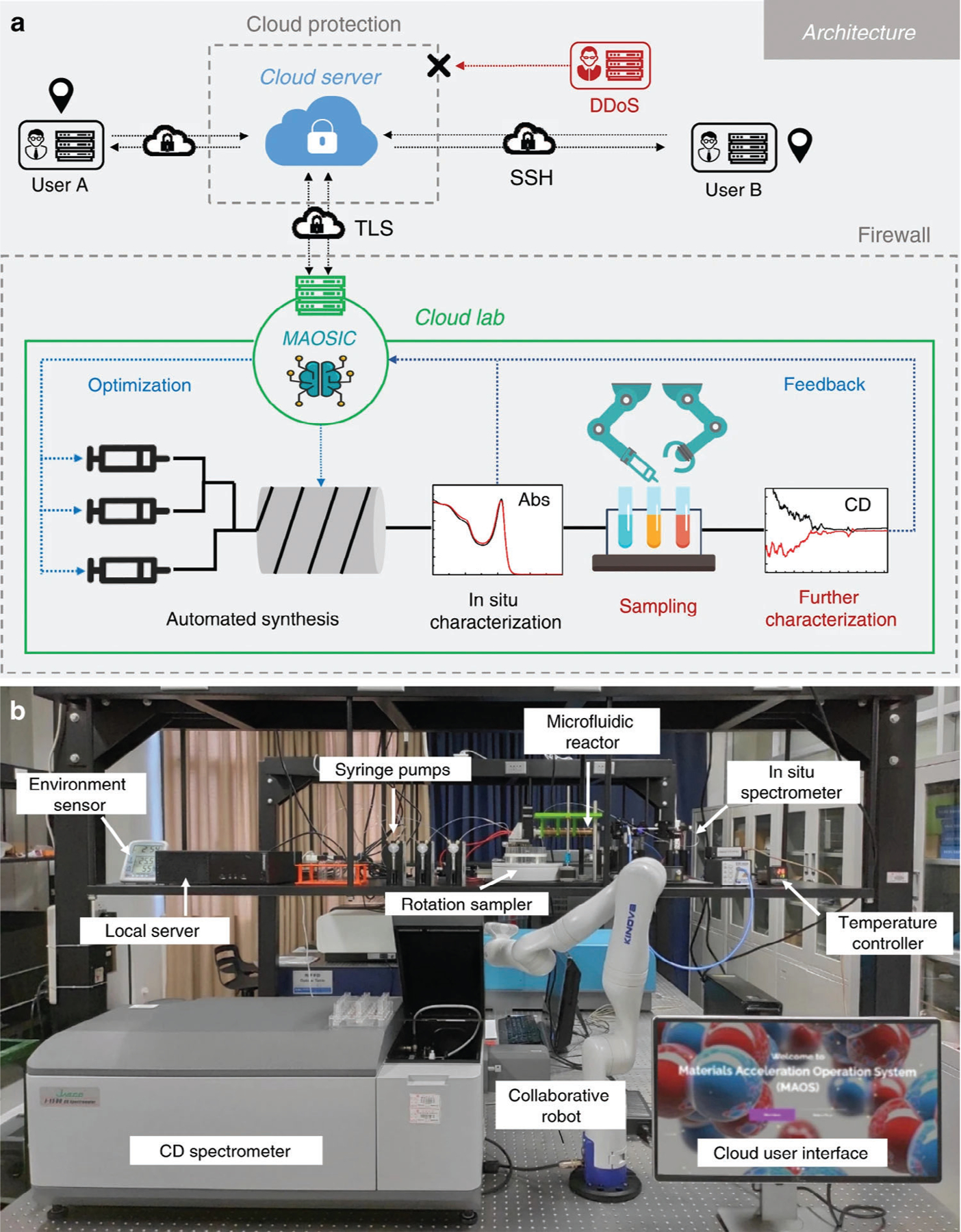
a) MAOSIC allows remote users to interact with the b) integrated equipment in the lab through the cloud server. Encrypted communication and firewalls were utilized to maintain data security and transfer stability. Automatic reactions took place in microfluidic reactors and under in situ monitoring through the absorption spectrum. Collaborative robots handled the automatic sampling task for CD measurement. All characterization data were sent back to the analysis and optimization module in MAOSIC for autonomous optimization. Reproduced with permission.^[349]^

Back in 2020, Li et al. described their development of what we will dub “the laboratory of the future”—a lab that integrates the technology of artificial intelligence of things, lab automation, and cloud servers, as shown in the schematic in Figure 10.^[349]^ An intelligent cloud lab facilitates the remote design of experiments to obtain materials with specified parameters, as well as remotely carrying out synthesis, characterization, parameter optimization, structural analysis, and theoretical calculations. Optically active inorganic perovskite NCs with temperature-dependent CD and inversion control were first synthesized using this intellectual platform. The thermodynamic mechanism for the formation of a chiral structure has also been theoretically studied.

A study from Liu et al. also details the use of an automated laboratory in combination with ML. An Authentic Intelligent Machine protocol, based on symbolic regression and NN, has been developed to describe the dielectric constant and lifetime of chiral-geometry systems in theory and was further extended to ligand-induced chiral optical activity for various chiral QDs in practice.^[281]^ Undoubtedly, the most recent and high-profile example of this level of laboratory automation came at the end of 2023 from Szymanski et al.^[350]^ Their laboratory setup dubbed the “A-Lab,” involved using ML models, trained on literature data, to predict synthetic procedures for the novel, theoretically stable materials, from Google DeepMind’s GNoME project.^[351]^ The material is then synthesized and characterized via XRD by a robotic arm, and the XRD analysis is conducted by ML models trained on data from Materials Project and the Inorganic Crystal Structure Database. Out of 58 potential targets, the authors claim to have successfully synthesized and characterized 41 novel materials over 17 days using the A-Lab. However, as experts in solid-state chemistry and crystallography have noted in the comments of the article, the ML-driven XRD analysis leaves a lot to be desired, raising questions on whether the target materials were even indeed successfully synthesized. While this work presents significant strides in ML-powered laboratory automation, and with extra materials characterization steps could be a game-changer in this area, it does seem as though it may be some time before “the laboratory of the future” is truly realized.

### 6.6. Sensing and Biomedical Applications

Widespread implementation of ML in materials science is anticipated to drive breakthroughs in the field of chiral sensing, and several recent reports may be indicative of the rise of this new area. Han et al. recently reported a ML-assisted design approach toward targeted chiral plasmonic sensors, enabled and optimized by a combination of genetic optimization and DL algorithms.^[352]^ Their developed algorithm could be used to tailor and enhance the chiroptical response and sensitivity of these sensors for targeted detection of specific chiral molecules. A recent study by Okur et al. detailed the production of a stereoselective “e-nose” for chiral sensing using chiral nanoporous MOFs.^[353]^ Employing a KNN model for data analysis, the chiral MOF-based sensors achieved a remarkable 96% accuracy in the differentiation of various chiral odor molecules and their enantiomers. A bright future is envisioned for the field of chiral sensing, driven in part by the advancement of ML.

An ML algorithm was developed to analyze the interaction of NPs with proteins based on the analysis of structural features contributing to the formation of NP–protein complexes.^[354]^ Graph-theoretical descriptors and geometrical descriptors, which both take into account chirality, were found to be uniformly applicable to biological and inorganic nanostructures. ML algorithms were trained on protein–protein interactions and then successfully applied for the prediction of NP-protein binding sites.

## 7. Conclusions and Future Outlook

In this review, we have explored the power of ML in the context of advancing nanotechnology. ML is an incredibly powerful technique in this regard, however so far it has been under-utilized for chiral nanomaterials applications, despite its huge potential. Collaboration between researchers in the fields of ML and chiral NMs is an exciting avenue that deserves to be explored.

Drawing inspiration from the successful experiences of applying ML for achiral NM, as discussed in this review, it is evident that this pool of knowledge can, and should, be extended to chiral nanomaterials. One of the important tasks that can be improved with ML is synthesis–structure–property–application relation, where for chiral materials the following parameters can be highlighted: i) type of chiral precursors used either during synthesis or post-synthesis treatment, reaction parameters, catalyst or chiral substrate, etc.; ii) presence of chiral defects in crystal structure, chemical composition, chiral shape, etc.; iii) circular dichroism signal, dissymmetry factor (*g*-factor), optical and catalytic responses, interaction with bio objects, etc. In addition, ML algorithms can facilitate signal processing, improving the signal-to-noise ratio and, for example, decreasing the limit of detection for sensing applications. The establishment of a correlation between these three groups of parameters for chiral materials is expected to support the optimization and prediction of morphology and chiroptical responses, along with the inverse task of deriving the synthesis parameters from properties of interest for chiral NMs. Thus, nanomaterials with high optical activity, CPL emission, and high enantioselectivity required for targeted applications can be designed using ML algorithms, and supported by experimental data and computer simulations which then will only need to be used for validation. Furthermore, when taking into consideration chiral material stability, toxicity, and cost-effectiveness, ML could be invaluable, as this falls completely within the capabilities of ML, given sufficient data.

Undoubtedly, it must be considered that to train any ML algorithm, a large amount of data is needed. In the ideal case, a high-quality database for chiral materials is all that is needed to accelerate the development of chiral NMs using ML. However, thus far there is no such database, which is another significant task for future research. Creating a high-quality database of chiral NMs is critical for the future development of this area, where the data meets all the requirements of quality (homogeneous, complete, accurate, consistent, sufficient, and structured). Furthermore, this data needs to be collected and compiled using standardized procedures to eliminate errors. The formation of high-quality and complete databases is an important task for the entire field of NMs as a whole. In other words, “ML needs data and numbers.”

However, even in the absence of such a database, interim strategies can be employed to stimulate ML-driven advancements in chiral NMs. First, extraction and compilation of data from published research on chiral NMs can be used to lay the foundations for a starting database, which can be incrementally added to, in order to create a more thorough, complete database as more results are published. AI can also be used to automatically extract data from published work. Additionally, the use of high-throughput microfluidic reactors will facilitate the generation of large amounts of experimental data in a short time, including data for chiral NMs. Furthermore, cloud-integrated laboratories combined with automated synthetic systems are expected to become more widespread, facilitating less resource-intensive and remote experimentation Finally, computer simulations also generate substantial amounts of data for ML analysis. For chiral NMs, the effect of chiral particle morphology or chiral assembly structure on CD spectra can be simulated, and subsequently used to train ML in structural optimization, to obtain desired *g*-factor values, and spectral features.

The strengths of ML in image enhancement, recognition, and classification can be transferred seamlessly to the analysis of chiral NMs. ML can be utilized in image analysis to differentiate between left- and right-handed enantiomers, to identify chiral defects via HRTEM, to enhance image resolution, and more. Moreover, ML algorithms will prove themselves to be invaluable in the analysis of spectral measurements with low signal-to-noise ratio and increasing detection limits in sensing. ML algorithms that have been developed, trained, and tested with achiral materials in mind can also be adapted for chiral NM, which in turn can be adapted to a wider range of tasks for chiral materials. Based on established algorithms, ready-to-use packages can be created, expanding access to ML tools for scientists unfamiliar with the “ins and outs” of ML. It is anticipated that many such software packages are expected to appear in the near future that can be used by material scientists to solve routine tasks and to develop novel materials. More progressive and advanced ML technologies can be brought into the field of study of NMs, and chiral NMs in particular. For example, while vision transformers have become the dominant DL method in other fields, their use in nanotechnology is still limited, despite their huge potential for image and spectroscopic analysis, mainly because transformers are usually trained on a much larger amount of data than is currently available for NMs. However, this problem can be solved by using such a method as transfer learning, i.e., the application of models pre-trained on datasets not related to chiral materials, and to nanomaterials in general, but adapted to a specific task related to chirality (fine-tuning). This approach has been applied to analyze spectrograms in other fields, such as audio and voice data, and can be applied to NM research.

To conclude, we believe that ML will play a pivotal role in shaping the future development of new chiral nanomaterials, offering unprecedented insights and accelerating progress in this exciting and rapidly progressing field. Chiral nanomaterials, with their unique asymmetric structures, hold immense promise for applications ranging from catalysis and drug delivery to electronics and photonics. However, designing and synthesizing these materials with precise chirality and desired properties remains a very complex challenge. By integrating data from experiments, simulations, and theoretical models, ML algorithms can uncover hidden correlations and will be hugely beneficial in helping scientists navigate the intricate world of chiral material design. As ML models continuously learn and improve from new data, they refine their predictions and adapt to evolving scientific understanding. Thus, in time, the predictive capability of ML should guide researchers toward the most promising chiral candidates, reducing the need for extensive experimentation and expediting the discovery of novel materials that exhibit specific chiroptical, catalytic, or electronic behaviors. We anticipate that ML will serve as a catalyst for innovation in the realm of chiral nanomaterials, fostering interdisciplinary collaboration, pushing the boundaries of our understanding, and shaping the field of materials science as a whole.

## Supplementary Material

Supplementary Material
