# Supplementary material for: Expanding the Horizons of Machine Learning in Nanomaterials to Chiral Nanostructures
Source: Adv Mater. Author manuscript; Available in PMC 2024 Jun 12. (PMC11167410; doi:10.1002/adma.202308912)
Supplement: Supplementary Material [file NIHMS1993426-supplement-Supplementary_Material.pdf]

ADVANCED MATERIALS

Supporting Information

for *Adv. Mater.*, DOI 10.1002/adma.202308912

Expanding the Horizons of Machine Learning in Nanomaterials to Chiral Nanostructures

Vera Kuznetsova, Áine Coogan, Dmitry Botov, Yulia Gromova, Elena V. Ushakova* and Yurii K. Gun'ko**

SUPPORTING INFORMATION

Expanding the Horizons of Machine Learning in Nanomaterials to Chiral Nanostructures

Vera Kuznetsova[1,*], *Áine Coogan*[1], *Dmitry Botov*[2,3], *Yulia Gromova*[4], *Elena V. Ushakova*[5,*], *Yori K. Gun'ko*[1,*]

1 - School of Chemistry, CRANN and AMBER Research Centres, Trinity College Dublin, College Green, Dublin, D02 PN40, Ireland.

2 - Everypixel Media Innovation Group, 021 Fillmore St., PMB 15, San Francisco, CA 94115, USA

3 – Neapolis University Pafos, 1 Danais Avenue, Pafos, 8042, Cyprus

4 - Department of Molecular and Cellular Biology, Harvard University, 52 Oxford St., Cambridge, MA 02138, USA

5 - Department of Materials Science and Engineering, and Centre for Functional Photonics (CFP), City University of Hong Kong, Hong Kong SAR 999077, P. R. China

Emails: kuznetsv@tcd.ie, eushakov@um.cityu.edu.hk, igounko@tcd.ie

Table S1. Available databases on materials and nanomaterials.

Name	*link*	*Number of records*	*Comments*
**Bulk materials databases**			
Materials Project; API available	materialsproject.org	154,718	Electronic structure, thermodynamics, mechanics, spectroscopy, synthesis
MPContribs; API available	mpcontribs.org	N/A	Electronic structure, thermodynamics, mechanics, surface info
NOMAD; API available	nomad-lab.eu	2,976,441	Electronic structure, thermodynamics, mechanics, spectroscopy
AFLOW; API available	aflowlib.org	3,530,330	Electronic structure, thermodynamics, mechanics
OQMD; API available	oqmd.org	1,022,603	Electronic structure, thermodynamics, DFT
Materials Cloud; API available	www.materialscloud.org	29,306,745	Electronic structure, thermodynamics, spectroscopy
NREL Materials Database	materials.nrel.gov	N/A	Electronic structure, thermodynamics, XRD
Computational Materials Repository	cmr.fysik.dtu.dk	N/A	Electronic structure, thermodynamics, mechanics, absorption spectra surface info
JARVIS; API available	jarvis.nist.gov	N/A	Modelling of properties by force-field, density functional theory, machine learning calculations; experimental data

MatCloud; API available	http://matcloud.cnic.cn		Electronic structure, thermodynamics, mechanics, surface info
MPDS; API available	mpds.io	1,6M	Electronic structure, thermodynamics, mechanics, spectroscopy, surface info
Materiae; API available	materiae.iphy.ac.cn	N/A	Electronic structure
Phonondb	phonondb.mtl.kyoto-u.ac.jp	N/A	Thermodynamics
MatNavi	mits.nims.go.jp	N/A	Electronic structure, thermodynamics, mechanics, XRD, surface info
Graph Networks for Materials Exploration (GNoME)	github.com/google-deepmind/materials_discovery	2,200,000	Electronic structure, thermodynamics, composition, crystal structure
**Nanomaterials databases**			
caNanoLab	https://cananolab.nci.nih.gov/	1573	Physico-chemical properties, in/ex vitro, clinical trial, protocols
S2NANO	http://portal.s2nano.org/	33,393	Physico-chemical properties, in vitro, cytotoxicity
eNanomapper	http://www.enanomapper.net/	N/A	Nanotoxicity
Nanomaterial registry	http://nanohub.org/ 10.1007/s11051-013-2219-8	N/A	Simulation tools and educational courses in nanotechnology, materials science, and related fields
DaNa Knowledge Base	https://www.nanopartikel.info/en/	N/A	Toxicology

NBI Knowledgebase	http://nbi.oregonstate.edu/	N/A	Characterization, synthesis protocols, nano-bio interaction, computational and data mining tools
Nanowerk	https://www.nanowerk.com/	5882	Nanotubes, Fullerenes, Graphene, Nanoparticles, Quantum Dots, Nanofibers, Nanowires
PubVINAS	http://www.pubvinas.com/	705	Physico-chemical properties, nano-bio interactions
NanoMine	https://materialsmine.org/nm	N/A	Materials genome prediction for polymer nanocomposites
DigiMOF	10.1021/acs.chemmater.3c00788	15,501	Metal-organic frameworks and their synthesis and properties
CMR	https://cmr.fysik.dtu.dk/c1db/c1db.html	3157	A systematic computational database for one-dimensional materials
CMR	https://cmr.fysik.dtu.dk/c2dm/c2dm.html#c2dm	52	Monolayer transition metal dichalcogenides and -oxides
CMR	https://cmr.fysik.dtu.dk/c2db/c2db.html	15,733	Computational 2D Materials Database (C2DB)
N/A	https://reedgroup.stanford.edu/databases.html	599 1D vdW solids and 1755 2D vdW solids	1D and 2D Materials Database

Table S2. Comparative parameters of ML methods for formation of database

ML algorithm	*Task, material*	*Training data size*	*Result and/or Reliability*	*Ref.*
Graph database	To understand the influence of aerogel synthetic and processing variables	997 aerogels from 97 manuscripts	103 aerogel instances with more than 7500 nodes and 20 000 edges	[1]
Supervised machine learning neural network regression model	Data cleaning for graph database: removing statistical outliers and prediction error; regression model of data	997 aerogels<br>Split of the dataset to 90% train and 10% test	Increasing an R2 value to 0.731, with MSE and RMSE values of 0.014 and 0.118, respectively	[1]
Assessment of data completeness and quality	Methodology to assess the completeness and quality of physicochemical and hazard datasets	81 860 entries	Establishment 11 physicochemical properties essential for data completeness assessment.<br>The quality of data was associated to classification of the data by used experimental protocols and/or by in vivo, in vitro and in silico studies.	[2]

Table S3. Comparative parameters of ML methods for Labelling and Augmentation

ML algorithm	*Task, material*	*Training data size*	*Result and/or Reliability*	*Ref.*
YOLOv3-style object detector and ResNet-152 neural networks	Figure separation, electron microscopy images of NMs	28962	Precision scores (F-score) of 0.92-0.98	[2]
Convolutional neural network: representation of images by hypercolumn vectors, clusterization by K-means, formation of Vector of Locally Aggregated Descriptors representation, training on the formed set the softmax classifier with gradient boosting algorithm	Classification of TEM images of carbon NM	5323 images	90.9% accuracy of classification for a 4-class dataset	[3]

Table S4. Comparative parameters of ML methods for Data Exploration

ML algorithm	*Task, material*	*Training data size*	*Result and/or Reliability*	*Ref.*
Linear discriminant analysis	Discrimination of silica NPs between engineered and natural	90 sample set was classified<br>Train/Test N/A	Accuracy of classification - 93.3%	[4]
Decision tree classifier (DTC), Random Forest (RF), Radial Basis Function Support Vector Machine (RBF SVM) and linear SVM	Classification of polymer nanocomposites by glass transition temperature	120 samples<br>Train/Test = 80/20	Accuracy: 0.852 (DTC), 0.922 (RF), 0.748 (RBF SVM), 0.757 (linear SVM)	[5]

Graph convolutional neural network	Classification of NMs by adsorption energies	N/A	N/A	[6]
principal component analysis, isometric mapping method, convolutional autoencoder	Data dimensionality reduction	N/A	N/A	[7]
Feature engineering				
SHapley Additive explanation	Identify model features to increase fit quality – 'atom contribution value' on example of C60 buckyball	384,260	The atoms with lowest contribution values in C60 are situated in the centre of the structure	[8]
SHapley Additive explanation with Random Forest	Identify nanodescriptors correlating with cell activation by gold NPs	46 samples	Identification of descriptors	[9]

Table S5. Comparative parameters of ML methods for Synthesis and structure – property correlation

ML algorithm	*Task, material*	*Training data size*	*Result and/or Reliability*	*Ref.*
Decision tree	Fe-Ni-Ti-Al alloy modelling – phase contents and critical temperatures	1815 data points<br>Train/Test = 70/30	R^2 = 0.986	[10]
K-nearest neighbour			R^2 = 0.994	
Adaptive boosting			R^2 = 0.975	
Random forest			R^2 = 0.998	

Spectral data pretreatment + random forest	Calibration model for laser-induced breakdown spectroscopy	750 spectra from 15 samples<br>Train/Test= 10/5	variable importance projection pretreatment + random forest R^2 = 0.9864<br>variable importance measurement + random forest R^2 = 0.9899<br>random forest R^2 = 0.9849	[11]
Principal component analysis and NN	Track transformations in CoxFe3-xO4 catalyst	20000 structure models	Principal component analysis of spectra revealed 4 spectroscopically distinct species. NN accuracy - N/A	[12]
gradient boosting decision tree	Set the dependence of vibrational density of states on Si structure at atomic level	Train/Test = 70/30	R^2 > 97%	[7]
Supervised NN	Dependence of electronic properties on conformational degrees of freedom of soft materials	Train samples - from approx. 2’000 to 11’000<br>Test samples - from approx. 25 to 225	R^2 increases with the dataset.<br>R^2 = 0.978 for SOMO energies, R^2 = 0.903 for spin energies	[13]
Dual variational autoencoder	Relation between SEM and scattering spectra or dark field images of gold NPs and clusters	898 clusters,<br>Train - 247 clusters,<br>Test - 8 clusters	Predicted geometry (SEM images) derived from dark field images are in a good agreement with initial SEM images	[14]

Multilevel attention graph convolution neural network (DeepMoleNet)<br>LightGBM	Prediction of surface energy and surface stress of hydroxyapatite nanoparticles from their structure	41,976 data samples<br>Train - 22’000<br>Test - 2’000	Light GBM based on DFT set R^2 = 0.96; DeepMoleNet based on DFTB set R^2 = 1.00; LightGBM based on CVFF set R^2= 0.93	[15]

Table S6. Comparative parameters of ML methods for Optimization and prediction

ML algorithm	*Task, material*	*Training data size*	*Result and/or Reliability*	*Ref.*
Deep neural network DeePMD-kit + active learning scheme Deep Potential Generator	Analysis of oxidation kinetics in MXenes	400 configurations and 11521 labelled data<br>Test - 1579<br>Train N/A	R^2>0.99	[16]
Decision tree (DT)<br>Random forest (RF)<br>Neural network	Chemical yield and crystallinity prediction for cellulose NCs	210 samples<br>Train/Test = 80/20	DT is better for crystallinity R^2 = 0.86.<br>RF is better for chemical yield R^2 = 0.89	[17]
Support vector regression model	Predict lattice constant and oxygen vacancy percentage of Ce-Zr-Mn catalyst	Train – 68 samples<br>Test – 8 samples	Lattice constant R^2=0.74<br>Oxygen vacancies R^2 = 0.76	[18]

Random forest (RF)<br>Gradient boosting (GB)<br>Extreme gradient boosting (XBM)<br>Support vector machine (SVM)	Prediction of topography – water drop adhesion on nano-structured surface	249 samples<br>Train/Test = 80/20	R^2 = 0.8531 (RF),<br>R^2 = 0.8397 (XGB),<br>R^2 = 0.8258 (GB),<br>R^2 = 0.8115 (SVM)	[19]
Graph convolutional NN	Prediction of silica aerogels surface area	approx.1000 samples<br>Train/Test = 90/10	R^2=0.731	[1]
Materials Graph Network	Data analysis of molecules and crystals with radius cutoff of 4A	130 462 molecules in the QM9 data set and 69 640 crystals in the Materials Project	Increased accuracy of prediction 11 target parameters out of 13	[20]
Materials Graph Network	Search for promising amines in the PubChem [10.1093/nar/gkaa971] database	5522 ionization potentials from quantum chemical computations	3 molecules were identified and experimentally validated	[21]

Table S7. Comparative parameters of ML methods for microscopic image processing

ML algorithm	*Task, material*	*Training data size*	*Result and/or Reliability*	*Ref.*
CNN	Phase determination of thin films of $Hf_{0.5}Zr_{0.5}O_2$	1075 images<br>Train/Test = 75/25	ResNet18 Accuracy of classification 95.82%	[22]
Transformers Enhanced Segmentation Network	NP size measurement	104 TEM images with 2169 NP<br>Train/Test = 344/72	Error of NP size ranges from 0.38-3.52%	[23]
Vision transformer	Automation of TEM images registration			[24]
TransMorph convolutional NN	Unsupervised medical image recording	Over 1000 MRI images	N/A	[25]

Table S8. Comparative parameters of ML methods for modelling using DFT and MD

ML algorithm	*Task, material*	*Training data size*	*Result and/or Reliability*	*Ref*
MALA NN	Extension of DFT prediction of electronic structures	N/A	mean absolute percentage error <7% for system of 2048 atoms	[26]
crystal graph convolutional NN	Estimation of energy gas adsorption for graphene-based catalyst	400 samples<br>Train/Test = 160/20	Model uncertainty 8-10% compared to DFT calculations	[27]

Table S9. Comparative parameters of ML methods for chiral NMs

ML algorithm	*Task, material*	*Training data type*	*Reliability*	*Ref.*
**Imaging**				
Deep learning model	identify the chiral morphology of twisted bowtie-shaped microparticles	SEM images		[28]
CNN	determine the handedness of chiral Tellurium NPs	SEM images		[29]
CNN	determine the chirality of CNTs	TEM images		[30]
CNN	automated determination of chirality of SWCNTs	HRTEM images	71% accuracy	[30]
U-net CNN	Determine of NP transport through tumour vessels	Optical microscopy images		[31]
	determine and spatially map the handedness of individual lanthanide-based nanocrystals	Optical microscopy images		[32]
**Modelling and simulation**				
NN trained on MD calculations	predict the mechanical properties of chiral single-walled carbon nanotubes		accurate predictions, comparable with DFT	[33]
CNN, fully connected neural network (FC-NN)	Metamaterials			[34] [34a]

**Prediction**				
DNN	mechanical properties of SWCNTs		dependence of the mechanical properties on θ with accuracy better than MD	[33]
ML model consisting of RF, ANN and SVM	examine the efficacy of using single-stranded DNA to sort SWCNTs according to their chirality		improving the success rate from 10% for the empirical approach to over 90%	[35]
Authentic Intelligent Machine (AIM) protocol, based on symbolic regression and neural network	describe the dielectric constant and lifetime of chiral-geometry systems in theory			[36]
Applications				
genetic optimisation and deep learning	tailor and enhance the chiroptical response and sensitivity of chiral plasmonic sensors			[37]
KNN	chiral sensing using chiral nanoporous MOFs		96% accuracy	[38]

Table S10. Comparative parameters of ML methods for chiral metamaterials

Metamaterial, structural unit	*Task*	*Simulation or measurement method*	*ML algorithms and tools*	*Results*	*Ref.*
Nanostructures, consisted of gold cuboids, placed on a SiO2/Si	Simulation: CD spectra. Experiment: CD sensing response for L and D-glucose	Simulation: finite-difference time-domain (FDTD), Experiment: Cathodoluminescence (CL) spectroscopy	NN, Reinforcement learning	MSE of training loss is 1.22×10−4, and the test loss is 3.31×10−4 ML-optimised structure was used for chiral sensing of L- and D-glucose	[39]
Vertically displaced and corner-stacked pair of gold nanorods (plasmonic Born–Kuhn model)	Circular differential extinction cross section which agrees with CD	FDTD	DL	Root-mean-square error (RMSE) 2.57 × 10–5	[37]
The shape of structural elements was widely varied	High-order diffracted CD	rigorous coupled-wave approach (RCWA) and CD data from alternative already studied MM (source domain dataset)	A model-agnostic data enhancement (MADE) including NN, RF and SV regression	Computational accuracy of NN is larger than 99%, one of SV is over 90%.	[34]
Structure consists of top yin-yang-shaped gold nanoparticles, a PMMA layer, a gold backreflector, and a bottom glass layer	CD spectra, electric-field distribution, Experiment: CD sensing	Finite element method (FEM)	Multitask DL, single bidirectional neural network with a joint-learning feature	MSE is 0.000441 ML-optimised structure was used for chiral sensing	[40]

Gold array of T-like shaped structures in the left handedness deposited on the oxidized silicon substrate	Third-order diffracted CD	RCWA	DNN: Fully connected neural network	MAE of 0.02 for the train dataset, whose value slightly increases to 0.03 for the test dataset, after the training process of 2000 epochs	[41]
Gold cubes with fixed dimensions (10 × 10 × 40 nm3) positioned on a 40 × 40 square matrix on the top of a Si=SiO2 substrate	Predict the electric-field distributio n, reflection and CD spectra	FDTD	Bayesian optimization (BO) and CNN, BoNet	The MSE after 100 epochs for electric-field prediction is 0.048 for training data and 0.068 for validation and testing data.<br><br>For reflection spectra, training loss is 1.30 × 10−4, validation loss is 3.52 × 10−4, and testing loss is 3.74 × 10−4.<br><br>Model was validated on real structures fabricated by E-beam lithography. Real CD was 82% of theoretically predicted at target wavelength	[42]

Two stacked gold split ring resonators twisted at a certain angle and separated by two spacing dielectric layers with a continuous gold reflector at the bottom	Predict the electric-field distribution and reflection and CD spectra	Numerical simulation package CST Microwave Studio is employed to generate the reflection spectra data by Monte Carlo sampling	DL model comprise two bidirectional neural networks: a primary network and an auxiliary network, assembled by a partial stacking strategy	Accuracy: mean square error (MSE) 0.00080	[43]

Commonly used abbreviations throughout the review

NMs - nanomaterials
NPs - nanoparticles
NCs - nanocrystals
QDs - quantum dots
MMs - metamaterials
SEM - scanning electron microscopy
TEM - transmission electron microscopy
AFM - atomic force microscopy
CNTs - carbon nanotubes
SWCNTs - single-walled carbon nanotubes
DFT - density functional theory
MD - molecular dynamics
FDTD - finite-difference time-domain
FEM - finite element method
AI - artificial intelligence
ML - machine learning

Brief descriptions of commonly used machine-learning-related terms throughout the review

ML

A program or system that trains a model from input data. The trained model can make useful predictions from new (never-before-seen) data drawn from the same distribution as the one used to train the model.

Machine learning also refers to the field of study concerned with these programs or systems. (https://developers.google.com/machine-learning/glossary#a)

Supervised ML

Training a model from features and their corresponding labels. Supervised machine learning is analogous to learning a subject by studying a set of questions and their corresponding answers. After mastering the mapping between questions and answers, a student can then provide answers to new (never-before-seen) questions on the same topic. (https://developers.google.com/machine-learning/glossary#a)

Unsupervised ML

Training a model to find patterns in a dataset, typically an unlabelled dataset. (https://developers.google.com/machine-learning/glossary#a)

AL - active learning

A training approach in which the algorithm chooses some of the data it learns from. Active learning is particularly valuable when labelled examples are scarce or expensive to obtain. Instead of blindly seeking a diverse range of labelled examples, an active learning algorithm selectively seeks the particular range of examples it needs for learning. (https://developers.google.com/machine-learning/glossary#a)

DL - deep learning

a class of machine learning algorithms that uses multiple layers to progressively extract higher-level features from the raw input.

VT - vision transformer

a transformer designed for computer vision. The basic structure is to break down input images as a series of patches, then tokenized, before applying the tokens to a standard Transformer architecture.

Neural networks, NN

Neural network, a model containing at least one hidden layer. A deep neural network is a type of neural network containing more than one hidden layer. For example, the following diagram shows a deep neural network containing two hidden layers. (https://developers.google.com/machine-learning/glossary#a)

ANN - artificial neural network
Neural networks implemented on computers are sometimes called *artificial neural networks* to differentiate them from neural networks found in brains and other nervous systems.(https://developers.google.com/machine-learning/glossary#a)

CNN - convolutional neural network
Neural network based on convolutional layers which are layers of a deep neural network in which a convolutional filter passes along an input matrix. (https://developers.google.com/machine-learning/glossary#a)

DNN - deep neural network
A neural network containing more than one hidden layer.

DenseNet - dense convolutional network
Network based on dense layers, hidden layers in which each node is connected to every node in the subsequent hidden layer. (https://developers.google.com/machine-learning/glossary#a)

RF - random forest
An ensemble of decision trees in which each decision tree is trained with a specific random noise, such as bagging. Random forests are a type of decision forest.

DT - decision tree
A supervised learning model composed of a set of conditions and leaves organized hierarchically.

Boosting algorithms
A machine learning technique that iteratively combines a set of simple and not very accurate classifiers (referred to as "weak" classifiers) into a classifier with high accuracy (a "strong" classifier) by upweighting the examples that the model is currently misclassifying. (https://developers.google.com/machine-learning/glossary#a)

Adaboost - adaptive boosting
a statistical classification meta-algorithm which can be used in conjunction with many other types of learning algorithms to improve performance. The output of the other learning algorithms ('weak learners') is combined into a weighted sum that represents the final output of the boosted classifier. AdaBoost is adaptive in the sense that subsequent weak learners are tweaked in favor of those instances misclassified by previous classifiers.

CatBoost - categorical boosting
an open-source software library which provides a gradient boosting framework which among other features attempts to solve for Categorical features using a permutation driven alternative compared to the classical algorithm.

SV - support vector
SVM - support vector machine
Supervised learning models with associated learning algorithms that analyze data for classification and regression analysis. Given a set of training examples, each marked as belonging to one of two categories, an SVM training algorithm builds a model that assigns new examples to one category or the other, making it a non-probabilistic binary linear classifier (although methods such as Platt scaling exist to use SVM in a probabilistic classification setting). SVM maps training examples to points in space so as to maximise the width of the gap between the two categories. New examples are then mapped into that same space and predicted to belong to a category based on which side of the gap they fall.

K-means
A popular clustering algorithm that groups examples in unsupervised learning. The k-means algorithm basically does the following: (i) iteratively determines the best k center points (known as centroids) (ii) assigns each example to the closest centroid. Those examples nearest the same centroid belong to the same group. The k-means algorithm picks centroid locations to minimize the cumulative square of the distances from each example to its closest centroid. (https://developers.google.com/machine-learning/glossary#a)

KNN - K-nearest-neighbour
k-NN is a type of classification where the function is only approximated locally and all computation is deferred until function evaluation. Since this algorithm relies on distance for classification, if the features represent different physical units or come in vastly different scales then normalizing the training data can improve its accuracy dramatically.

SHAP - Shapley Additive exPlanations
It is based on Shapley values, which use game theory to assign credit for a model's prediction to each feature or feature value. The way SHAP works is to decompose the output of a model by the sums of the impact of each feature. SHAP calculates a value that represents the contribution of each feature to the model outcome. (https://datascientest.com/en/shap-what-is-it#:~:text=SHapley%20Additive%20exPlanations%2C%20more%20commonly,each%20feature%20or%20feature%20value)

PCA - principal component analysis
is a popular technique for analyzing large datasets containing a high number of dimensions/features per observation, increasing the interpretability of data while preserving the maximum amount of information, and enabling the visualization of multidimensional data. Formally, PCA is a statistical technique for reducing the dimensionality of a dataset. This is accomplished by linearly transforming the data into a new coordinate system where (most of) the variation in the data can be described with fewer dimensions than the initial data.

LDA - linear discriminant analysis

normal discriminant analysis (NDA), or discriminant function analysis is a generalization of Fisher's linear discriminant, a method used in statistics and other fields, to find a linear combination of features that characterizes or separates two or more classes of objects or events. The resulting combination may be used as a linear classifier, or, more commonly, for dimensionality reduction before later classification.

References

[1] R. C. Walker, A. P. Hyer, H. Guo, J. K. Ferri, *Chemistry of Materials* **2023**, *35*, 4897-4910.

[2] G. Basei, H. Rauscher, N. Jeliazkova, D. Hristozov, *Nanotoxicology* **2022**, *16*, 195-216.

[3] Q. Luo, E. A. Holm, C. Wang, *Nanoscale Advances* **2021**, *3*, 206-213.

[4] X. Yang, X. Liu, A. Zhang, D. Lu, G. Li, Q. Zhang, Q. Liu, G. Jiang, *Nature Communications* **2019**, *10*, 1620.

[5] B. Ma, N. J. Finan, D. Jany, M. E. Deagen, L. S. Schadler, L. C. Brinson, *Macromolecules* **2023**, *56*, 3945-3953.

[6] K. Bang, D. Hong, Y. Park, D. Kim, S. S. Han, H. M. Lee, *Nature Communications* **2023**, *14*, 3004.

[7] D. Vizoso, G. Subhash, K. Rajan, R. Dingreville, *Chemistry of Materials* **2023**, *35*, 1186-1200.

[8] A. S. Anker, E. T. S. Kjær, M. Juelsholt, T. L. Christiansen, S. L. Skjærvø, M. R. V. Jørgensen, I. Kantor, D. R. Sørensen, S. J. L. Billinge, R. Selvan, K. M. Ø. Jensen, *npj Computational Materials* **2022**, *8*, 213.

[9] J. Ma, S. Wang, C. Zhao, X. Yan, Q. Ren, Z. Dong, J. Qiu, Y. Liu, Q. e. Shan, M. Xu, B. Yan, S. Liu, *Angewandte Chemie International Edition* **2023**, *62*, e202301059.

[10] C. Tan, Q. Li, X. Yao, L. Chen, J. Su, F. L. Ng, Y. Liu, T. Yang, Y. Chew, C. T. Liu, T. DebRoy, *Advanced Science* **2023**, *10*, 2206607.

[11] T. Chen, T. Zhang, H. Tang, X. Cheng, H. Li, *Analytical Chemistry* **2023**, *95*, 4819-4827.

[12] J. Timoshenko, F. T. Haase, S. Saddeler, M. Rüscher, H. S. Jeon, A. Herzog, U. Hejral, A. Bergmann, S. Schulz, B. Roldan Cuenya, *Journal of the American Chemical Society* **2023**, *145*, 4065-4080.

[13] R. Alessandri, J. J. de Pablo, *Macromolecules* **2023**, *56*, 3574-3584.

[14] M. Y. Yaman, S. V. Kalinin, K. N. Guye, D. S. Ginger, M. Ziatdinov, *Small* **2023**, *19*, 2205893.

[15] Z. Liu, Y. Shi, H. Chen, T. Qin, X. Zhou, J. Huo, H. Dong, X. Yang, X. Zhu, X. Chen, L. Zhang, M. Yang, Y. Gao, J. Ma, *npj Computational Materials* **2021**, *7*, 142.

[16] P. Hou, Y. Tian, Y. Xie, F. Du, G. Chen, A. Vojvodic, J. Wu, X. Meng, *Angewandte Chemie International Edition* **2023**, *62*, e202304205.

[17] H. Wang, Q. Du, Y. Liu, S. Cheng, *Cellulose* **2023**, *30*, 6273-6287.

[18] C. Wang, B. Ko, M. Najimu, E. Sasmaz, *Chemistry of Materials* **2023**, *35*, 1926-1934.

[19] X. He, K. Zhang, X. Xiong, Y. Li, X. Wan, Z. Chen, Y. Wang, X. Xu, M. Liu, Y. Jiang, S. Wang, *Small* **2022**, *18*, 2203264.

[20] C. Chen, W. Ye, Y. Zuo, C. Zheng, S. P. Ong, *Chemistry of Materials* **2019**, *31*, 3564-3572.

[21] Z. Mao, C. Chen, Y. Zhang, K. Suzuki, Y. Suzuki, *Advanced Materials*, *n/a*, 2303827.

[22] Z. Cheng, X. Xie, Y. Yang, C. Wang, C. Luo, H. Bi, Y. Wang, J. Chu, X. Wu, *Materials Today Electronics* **2023**, *3*, 100027.

[23] Z. Wang, L. Fan, Y. Lu, J. Mao, L. Huang, J. Zhou, *Powder Technology* **2022**, *407*, 117673.

[24] F. Feng, T. Zhang, R. Sun, J. He, Z. Xiong, F. Wu, in *Neural Information Processing: 29th International Conference, ICONIP 2022, Virtual Event, November 22–26, 2022, Proceedings, Part III*, Springer-Verlag, New Delhi, India, **2023**, pp. 14–25.

[25] J. Chen, E. C. Frey, Y. He, W. P. Segars, Y. Li, Y. Du, *Med Image Anal* **2022**, *82*, 102615.

[26] L. Fiedler, N. A. Modine, S. Schmerler, D. J. Vogel, G. A. Popoola, A. P. Thompson, S. Rajamanickam, A. Cangi, *npj Computational Materials* **2023**, *9*, 115.

[27] K. Boonpalit, Y. Wongnongwa, C. Prommin, S. Nutanong, S. Namuangruk, *ACS Applied Materials & Interfaces* **2023**, *15*, 12936-12945.

[28] A. Visheratina, A. Visheratin, P. Kumar, M. Veksler, N. A. Kotov, *ACS Nano* **2023**, *17*, 7431-7442.

[29] C. K. Groschner, A. J. Pattison, A. Ben-Moshe, A. P. Alivisatos, W. Theis, M. C. Scott, *npj Computational Materials* **2022**, *8*, 149.

[30] G. D. Förster, A. Castan, A. Loiseau, J. Nelayah, D. Alloyeau, F. Fossard, C. Bichara, H. Amara, *Carbon* **2020**, *169*, 465-474.

[31] M. Zhu, J. Zhuang, Z. Li, Q. Liu, R. Zhao, Z. Gao, A. C. Midgley, T. Qi, J. Tian, Z. Zhang, D. Kong, J. Tian, X. Yan, X. Huang, *Nat Nanotechnol* **2023**, *18*, 657-666.
[32] E. Vinegrad, U. Hananel, G. Markovich, O. Cheshnovsky, *ACS Nano* **2019**, *13*, 601-608.
[33] M. Čanađija, *Carbon* **2021**, *184*, 891-901.
[34] *a)*H. S. Khaliq, A. Nauman, J.-W. Lee, H.-R. Kim, *Advanced Optical Materials* **2023**, *11*, 2300644; *b*)S. Du, J. You, J. Zhang, Z. Tao, H. Hao, Y. Tang, X. Zheng, T. Jiang, *Nanophotonics* **2021**, *10*, 1155-1168.
[35] Z. Lin, Y. Yang, A. Jagota, M. Zheng, *ACS Nano* **2022**, *16*, 4705-4713.
[36] R. Liu, J. Li, S. Xiao, D. Zhang, T. He, J. Cheng, X. Zhu, *ACS Nano* **2022**, *16*, 1600-1611.
[37] J. H. Han, Y.-C. Lim, R. M. Kim, J. Lv, N. H. Cho, H. Kim, S. D. Namgung, S. W. Im, K. T. Nam, *ACS Nano* **2023**, *17*, 2306-2317.
[38] S. Okur, P. Qin, A. Chandresh, C. Li, Z. Zhang, U. Lemmer, L. Heinke, *Angewandte Chemie International Edition* **2021**, *60*, 3566-3571.
[39] Y. Chen, F. Zhang, Z. Dang, X. He, C. Luo, Z. Liu, P. Peng, Y. Dai, Y. Huang, Y. Li, Z. Fang, *Opto-Electronic Science* **2023**, *2*, 220019-220011-220019-220010.
[40] E. Ashalley, K. Acheampong, L. V. Besteiro, P. Yu, A. Neogi, A. O. Govorov, Z. M. Wang, *Photon. Res.* **2020**, *8*, 1213-1225.
[41] Z. Tao, J. Zhang, J. You, H. Hao, H. Ouyang, Q. Yan, S. Du, Z. Zhao, Q. Yang, X. Zheng, T. Jiang, *Nanophotonics* **2020**, *9*, 2945-2956.
[42] Y. Li, Y. Xu, M. Jiang, B. Li, T. Han, C. Chi, F. Lin, B. Shen, X. Zhu, L. Lai, Z. Fang, *Physical Review Letters* **2019**, *123*, 213902.
[43] W. Ma, F. Cheng, Y. Liu, *ACS Nano* **2018**, *12*, 6326-6334.
